# Modulation of Dendritic Cell Function via Nanoparticle-Induced Cytosolic Calcium Changes

**DOI:** 10.1021/acsnano.4c00550

**Published:** 2024-02-29

**Authors:** Zhengwei Cao, Xueyuan Yang, Wei Yang, Fanghui Chen, Wen Jiang, Shuyue Zhan, Fangchao Jiang, Jianwen Li, Chenming Ye, Liwei Lang, Sirui Zhang, Zhizi Feng, Xinning Lai, Yang Liu, Leidong Mao, Houjian Cai, Yong Teng, Jin Xie

**Affiliations:** † Department of Chemistry, University of Georgia, Athens, Georgia 30602, United States; ‡ Department of Hematology and Medical Oncology & Winship Cancer Institute, Emory University School of Medicine, Atlanta, Georgia 30322, United States; § Department of Pharmaceutical and Biomedical Sciences, College of Pharmacy, University of Georgia, Athens, Georgia 30602, United States; ∥ Department of Physiology, Medical College of Georgia, Augusta University, Augusta, Georgia 30907, United States; ⊥ Institute of Bioinformatics, University of Georgia, Athens, Georgia 30602, United States; # School of Electrical and Computer Engineering, College of Engineering, University of Georgia, Athens, Georgia 30602, United States

**Keywords:** cancer, calcium, nanoparticles, immunotherapy, dendritic cells, radiotherapy

## Abstract

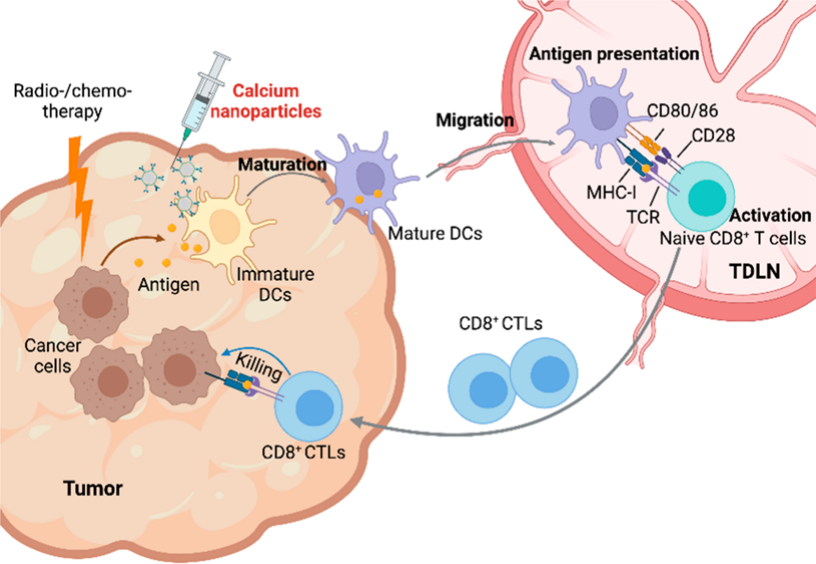

Calcium nanoparticles have been investigated for applications, such as drug and gene delivery. Additionally, Ca^2+^ serves as a crucial second messenger in the activation of immune cells. However, few studies have systematically studied the effects of calcium nanoparticles on the calcium levels and functions within immune cells. In this study, we explore the potential of calcium nanoparticles as a vehicle to deliver calcium into the cytosol of dendritic cells (DCs) and influence their functions. We synthesized calcium hydroxide nanoparticles, coated them with a layer of silica to prevent rapid degradation, and further conjugated them with anti-CD205 antibodies to achieve targeted delivery to DCs. Our results indicate that these nanoparticles can efficiently enter DCs and release calcium ions in a controlled manner. This elevation in cytosolic calcium activates both the NFAT and NF-κB pathways, in turn promoting the expression of costimulatory molecules, antigen-presenting molecules, and pro-inflammatory cytokines. In mouse tumor models, the calcium nanoparticles enhanced the antitumor immune response and augmented the efficacy of both radiotherapy and chemotherapy without introducing additional toxicity. Our study introduces a safe nanoparticle immunomodulator with potential widespread applications in cancer therapy.

Dendritic cells (DCs) are the most effective type of antigen presenting cells (APCs) and play an essential role in protection against malignancies.^1−3^ DCs constitutively sample their surroundings for antigens, process them, and then migrate to the secondary lymphoid where they prime naïve T cells.^3^ During this process, DCs undergo maturation, marked by the upregulation of antigen-presenting molecules like MHC-II^4,5^ and costimulatory molecules such as CD80, CD86, and CCR7.^4,5^ Meanwhile, DCs also secrete cytokines including interleukin-12 (IL-12) and type I interferons, which shape T cell responses.^6,7^ As such, DCs serve as a critical bridge between innate and adaptive immune responses.

However, the tumor microenvironment (TME) is often rich in immunosuppressive factors^8−11^ that act on DCs to dampen the immunity or induce tolerance.^4,12^ The implications are far-reaching, as many cancer treatments depend on DC-mediated immunity. For example, while the focus of radiation therapy has been on causing DNA damage to induce cell apoptosis or mitotic catastrophe, recent studies have shown that the radiation-induced immune response also plays an important role.^13,14^ This encompasses radiation-induced release of tumor-associated antigens, secretion of type-1 interferons, and upregulation of antigen-presenting molecules, which foster DC maturation and cross-presentation. Similarly, chemotherapy has been shown to induce immunogenic cell death (ICD),^15^ marked by the release of danger-associated molecular patterns (DAMPs) that stimulate DC maturation.^16,17^ On the other hand, the lack of activated DCs in tumors can significantly impede immunotherapy.^18,19^ Adding adjuvants that invigorate DCs could boost immune reactions and enhance therapeutic outcomes.^19−21^ However, conventional adjuvants such as cytokines, poly(I:C), and lipopolysaccharide (LPS) are often associated with problems such as rapid clearance, off-target toxicity, and suboptimal efficacy, which have limited their use in the clinic.

Ca^2+^ as a second messenger plays an important role in the maturation and migration of DCs.^22^ Resting, immature DCs maintain a low level of cytosolic calcium or [Ca^2+^]_int_.^22^ Cytokines, pathogen-associated molecular patterns, or DAMPs can bind to DC receptors, initiating a signaling cascade that causes calcium release from the endoplasmic reticulum (ER) into the cytosol, followed by calcium influx via the Ca^2+^ release-activated Ca^2+^ (CRAC) channels.^23−27^ This increase in [Ca^2+^]_int_ activates signaling pathways including the NFAT and NF-KB pathways, promoting expression of costimulatory and antigen-presenting molecules, thereby facilitating DC maturation.^22,26^ An artificially induced elevation in [Ca^2+^]_int_, i.e., direct delivery of calcium into the cytosol, may activate DCs, a speculation that has been confirmed *in vitro* with calcium ionophores such as ionomycin.^28^ However, DC maturation and activation requires a sustained increase in [Ca^2+^]_int_.^29^ The rapid clearance and lack of specificity of conventional calcium ionophores have prevented their *in vivo* applications. There is an unmet need for safe and effective calcium modulators that can enhance the DC-mediated anticancer immunity.

Here, we investigate calcium nanoparticles as an immunomodulator that can target DCs and modulate their cytosolic calcium levels, which in turn affect DC functions. We speculate that calcium nanoparticles can be internalized by DCs via endocytosis and release calcium therein to elevate [Ca^2+^]_int_, thereby promoting DC maturation, migration, and cross-presentation, in turn augmenting T cell immunity (Figure S1). To this end, we coated calcium nanoparticles with a layer of silica to allow the controlled release of calcium. In addition, we coupled anti-CD205 antibodies to the silica coating to enable the selective delivery of the nanoparticles to DCs. Calcium nanoparticles have been widely investigated as gene or drug delivery vehicles in the past^30,31^ and more recently as a means to modulate the pH of the TME.^32^ However, to the best of our knowledge, there has been no effort to systematically assess their influence on the intracellular calcium levels of DCs and the resultant effects on their functions. Their role as a relatively biocompatible immunomodulator and adjuvant has not been investigated either. We test our hypothesis first *in vitro* with mouse bone marrow-derived dendritic cells (BMDCs) and then *in vivo* as an adjuvant to RT or chemotherapy.

## Results and Discussion

### Synthesis, Surface Modification, and Physicochemical Characterization of Nanoparticles

**Figure 1:**
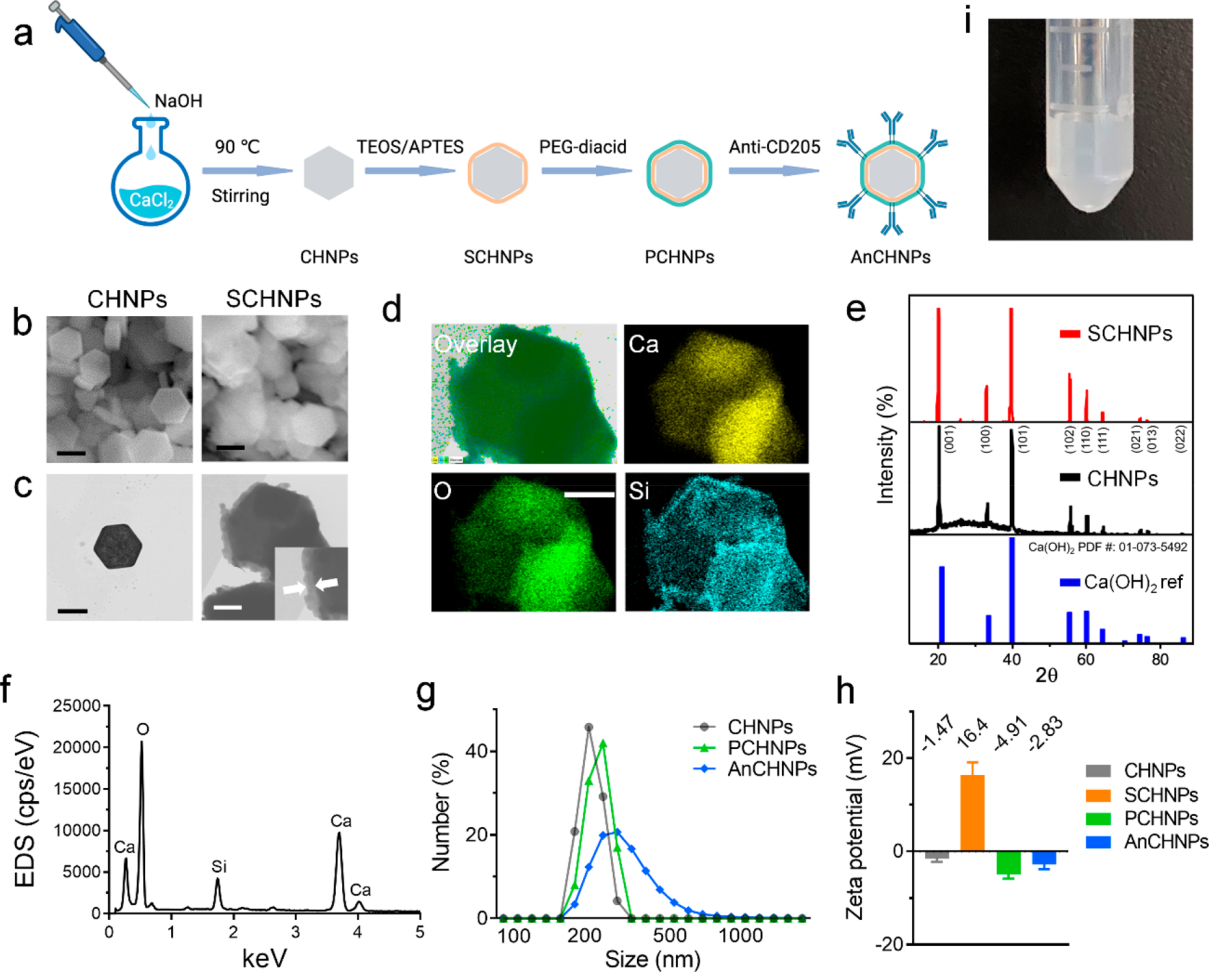
Synthesis and characterization of AnCHNPs. (a) Schematic illustration showing the steps of nanoparticle synthesis, surface coating, and antibody conjugation. (b) SEM images of CHNPs and SCHNPs. Scale bars, 200 nm. (c) TEM images of CHNPs (left) and SCHNPs (right). Scale bars: 200 nm (black) and 100 nm (white), respectively. (d) EDS elemental mapping shows the core/shell structure of SCHNPs. Scale bar, 250 nm. (e) XRD spectra of SCHNPs, CHNPs, and the Ca(OH)_2_ reference. (f) EDS spectra of SCHNPs. (g) DLS spectra of CHNPs, PCHNPs, and AnCHNPs, tested in water. (h) Zeta potentials of CHNPs, SCHNPs, PCHNPs, and AnCHNPs, measured in PBS (*n* = 3). (i) AnCHNPs are stable in PBS after incubation for 24 h.

Calcium hydroxide nanoparticles (CHNPs) were synthesized by a coprecipitation method using CaCl_2_ and NaOH as precursors (Figure 1a). Scanning electron microscopy (SEM) (Figure 1b) and transmission electron microscopy (TEM) (Figure 1c,d) show that the CHNPs are hexagonal in shape, with an average diameter (the long diagonal of the hexagons) of 219.9 ± 17.8 nm. X-ray powder diffraction (XRD) confirms that the nanocrystals are hexagonal Ca(OH)_2_ (PDF # 01–073–5492, Figure 1e).

The CHNPs were then coated with silica (Figure 1a). We used a mixture of tetraethyl orthosilicate (TEOS) and (3-aminopropyl)triethoxysilane (APTES) as silane precursors, so that the resulting nanoparticles have amine groups on the surface. We then conjugated polyethylene glycol (PEG) diacid (m.w. = 2000) to the silica surface by EDC/NHS coupling. SEM and energy dispersive spectroscopy (EDS) confirm the successful coating (Figure 1b,d,f). TEM shows that the coating thickness is ∼20 nm (Figure 1c). XRD confirms that the coating does not negatively affect the crystallinity of the Ca(OH)_2_ core (Figure 1e).

The PEGylated Ca(OH)_2_/SiO_2_ core–shell nanoparticles (PCHNPs) are well dispersed in water. Their hydrodynamic size is 245.2 ± 30.26 nm, compared to 227.3 ± 27.02 nm for bare Ca(OH)_2_/SiO_2_ nanoparticles (Figure 1g). The surface of the PCHNPs is almost neutral (−4.91 mV, Figure 1h). As a comparison, bare Ca(OH)_2_/SiO_2_ nanoparticles are slightly positively charged (+16.4 mV) due to surface amine groups. Successful PEGylation was also confirmed by Fourier transform infrared (FT-IR), which found characteristic stretching (2882 cm^–1^) and bending (1467 and 1341 cm^–1^) peaks of C–H, as well as the C–O–C stretching peak (1033 cm^–1^) with PCHNPs (Figure S2).

Finally, we coupled an anti-CD205 antibody to PCHNPs using EDC/NHS chemistry. CD205, also known as DEC205, is a type I integral membrane protein expressed primarily on DCs.^33^ The resulting conjugates, i.e., AnCHNPs, were stable in aqueous solutions (Figure 1i). Based on protein and calcium quantification, it is estimated that each nanoparticle carries on average 27 antibody molecules. Upon coupling with antibodies, the hydrodynamic size of the nanoparticles increased to 295.3 ± 46.7 nm (Figure 1g). Meanwhile, the surface charge was slightly increased to −2.83 mV over the conjugation (Figure 1h, Figure S2).

In summary, we have synthesized Ca(OH)_2_ nanoparticles, coated them with silica, and PEGylated the surface. We then successfully conjugated anti-CD205 antibodies to the nanoparticles.

### Uptake of AnCHNPs by DCs and Its Effect on [Ca^2+^]_int_

**Figure 2:**
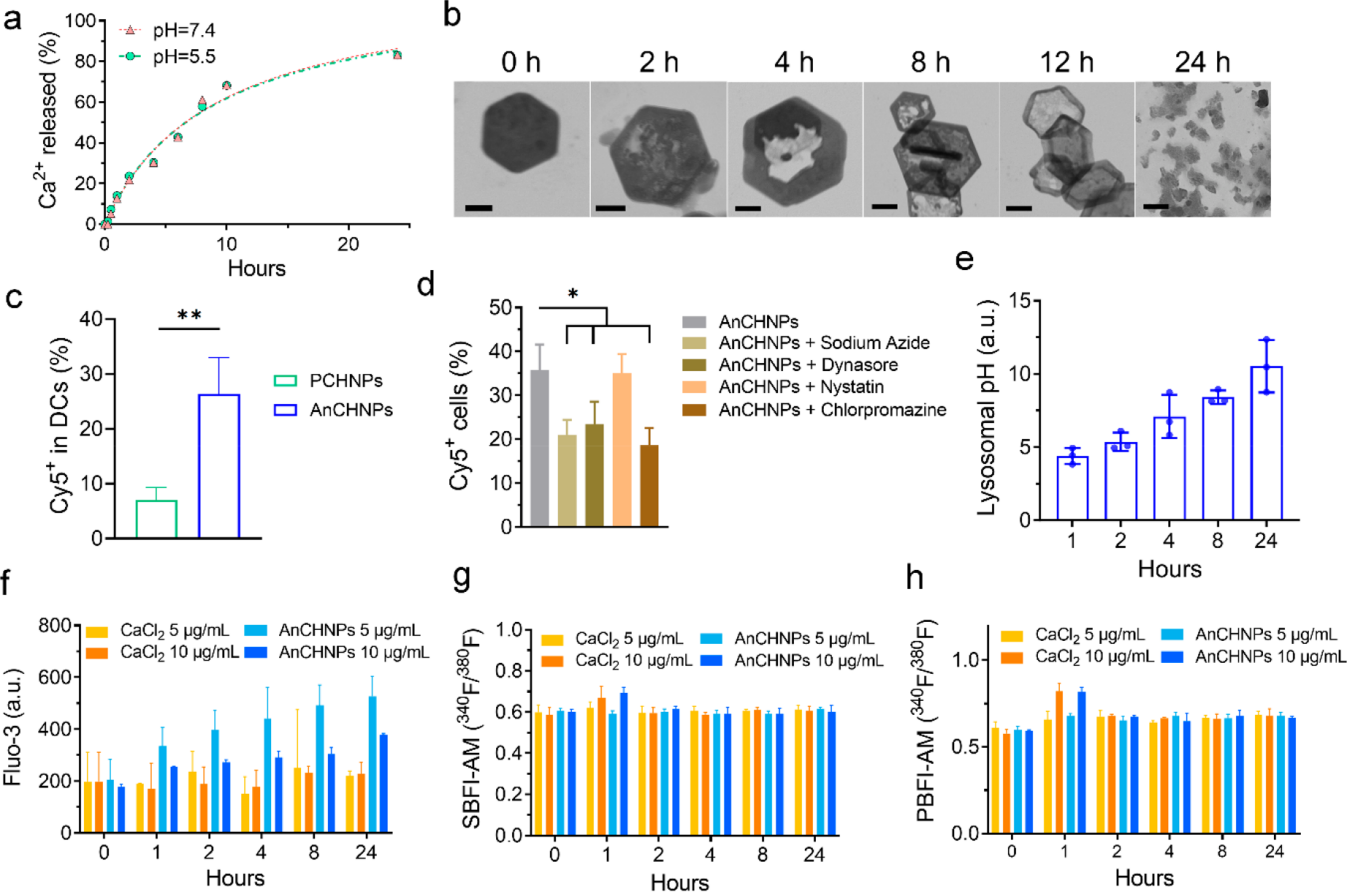
Stability and degradation of AnCHNPs. (a) Time-dependent release of Ca^2+^ from PCHNPs, tested in ammonium acetate buffer solutions at pH 7.4 and 5.5. (b) TEM images of PCHNPs showing that the calcium core was gradually degraded during incubation in water. Scale bars: 100 nm. (c) DC uptake of AnCHNPs (Cy5-labeled, 5 μg/mL). Compared to PCHNPs, AnCHNPs showed significantly increased cellular uptake (*n* = 3). (d) DC uptake when AnCHNPs were coincubated with endocytosis inhibitors including sodium azide (50 mM), dynasore (80 μM), nystatin (25 μM), and chlorpromazine (100 μM) (*n* = 3). (e) DC lysosomal pH change when the cells were incubated with AnCHNPs (5 μg/mL) (*n* = 3). (f) DC [Ca^2+^]_int_ changes when the cells were treated with AnCHNPs or CaCl_2_ (5 or 10 μg/mL), assessed by Fluo-3 AM (*n* = 3). (g) [Na^+^]_int_ of DCs when the cells were incubated with AnCHNPs or CaCl_2_ (5 or 10 μg/mL), assessed by SBFI-AM (*n* = 3). (h) [K^+^]_int_ of DCs when the cells were incubated with AnCHNPs or CaCl_2_ (5 or 10 μg/mL), assessed by PBFI-AM (*n* = 3). Data are presented as the mean ± s.d. *, *p* < 0.05.

The silica coating slows but does not prevent the degradation of the Ca(OH)_2_ core. We observed a sustained calcium release from PCHNPs in buffer solutions at neutral pH (Figure 2a, Figure S3). The cumulative release reached ∼80% after 24 h (Figure 2a), and the degradation rate hardly changed when the pH was lowered to 5.5, indicating that the nanoparticle degradation is dominated by water dissolution rather than the acid–base reaction. We also examined samples taken from PCHNPs solutions at different times under TEM. Consistent with the release results, there was a gradual dissolution of the Ca(OH)_2_ core (Figure 2b). Meanwhile, the silica shell remained largely intact during the first 12 h, effectively acting as a capsule for calcium.

We then examined the cellular uptake of the AnCHNPs by BMDCs. We labeled AnCHNPs with Cy5 and incubated the particles with BMDCs at 5 or 10 μg/mL (concentration based on calcium, the same as below). For comparison, we also tested Cy5-labeled PCHNPs (not conjugated with the antibody). Flow cytometry revealed significantly increased nanoparticle uptake with AnCHNPs compared to PCHNPs (Figure 2c). Uptake was reduced when AnCHNPs were coincubated with azide, a general inhibitor of endocytosis. Internalization was also inhibited by chlorpromazine and dynasore (Figure 2d), which block clathrin- and dynamin-dependent endocytosis, respectively. Meanwhile, nystatin, which inhibits the caveolae endocytosis pathway, had no effect on particle uptake. These results suggest that AnCHNPs enter DCs through receptor-mediated endocytosis, which has been observed by others using anti-CD205 antibodies.^34,35^

The LysoSensor assay shows that incubation with AnCHNPs caused an increase in the lysosomal pH of DCs (Figure 2e, Figure S3), which is attributed to proton neutralization by Ca(OH)_2_. Meanwhile, the Fluo-3AM assay revealed a time-dependent increase in the level of [Ca^2+^]_int_ (Figure 2f). This is due to the degradation of Ca(OH)_2_ particles and the parallel release of calcium into the cytosol. The increase in [Ca^2+^]_int_ persisted for more than 24 h, which is consistent with what was observed in the solutions. In comparison, CaCl_2_ salt induced little increase in [Ca^2+^]_int_ at the same calcium doses (Figure 2f). Meanwhile, [Na^+^]_int_ and [K^+^]_int_ levels remained unchanged during the nanoparticle incubation according to SBFI-AM and PBFI-AM assays (Figure 2g,h).

Taken together, our results confirmed that AnCHNPs are taken up by DCs through clathrin- and dynamin-dependent endocytosis and are gradually degraded inside the cells to allow for a sustained increase in [Ca^2+^]_int_.

### Effect of AnCHNPs on DC Maturation and Migration

**Figure 3:**
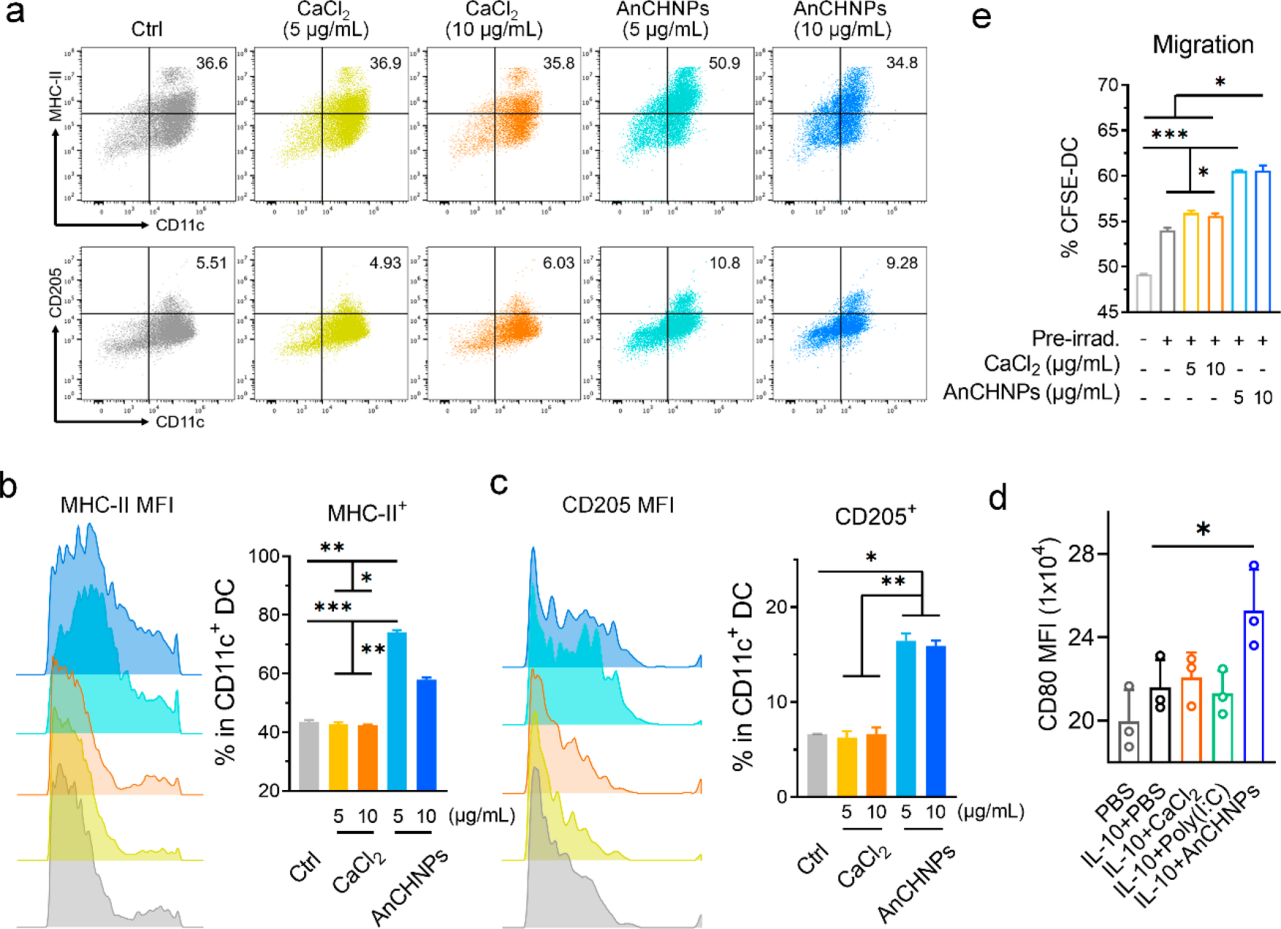
Impact of AnCHNPs on DC maturation and migration, tested with BMDCs. DCs were incubated with AnCHNPs or CaCl_2_ (5 or 10 μg/mL). (a–c) Populations and mean fluorescence intensities (MFIs) of MHC-II^+^ and CD205^+^ cells (*n* = 3). (a) Quadrants showing the changes of MHC-II^+^ and CD205^+^ populations among DCs (CD11^+^). Histograms showing the MFI and population changes for (b) MHC-II^+^ and (c) CD205^+^ cells. (d) Transwell assay examining DC migration. B16F10-OVA cells with or without preirradiation (100 Gy) were seeded into the bottom chamber, while CFSE-labeled DCs were loaded into the inset. CFSE^+^ cells in the lower chamber at 24 h were quantified by flow cytometry (*n* = 3). (e) MFIs of CD80 on DCs when incubated with IL-10 (*n* = 3). Data are presented as the mean ± s.d. *, *p* < 0.05; **, *p* < 0.01; ***, *p* < 0.001.

We first incubated AnCHNPs with BMDCs at 5 or 10 μg/mL in the absence of cancer cells and analyzed surface MHC-II by flow cytometry (Figure 3a). Compared to untreated DCs, both the population of MHC-II^+^ DCs and the expression level of MHC-II were significantly increased over the incubation time (Figure 3b), suggesting enhanced DC maturation. AnCHNPs also induced CD205 expression in DCs (Figure 3c). This is consistent with observations by others that CD205 is upregulated in activated DCs.^36^ The upregulation of CD205 creates a positive feedback loop that further promotes the uptake of the AnCHNPs and cell maturation. In comparison, CaCl_2_ had no effect on either MHC-II or CD205 expression (Figure 3a-c). Aged AnCHNPs (i.e., silica shell) also had no positive effect on MHC-II expression (Figure S4). Note that AnCHNPs were more effective at 5 μg/mL than 10 μg/mL, which is due to some degree of toxicity at higher pH (Figure S3c). We additionally tested the impact of AnCHNPs when DCs were coincubated with IL-10, which is able to blunt DC maturation and antigen-presentation.^37^ We found that AnCHNPs promote DC maturation despite the immunosuppressive environment (Figure 3d), whereas calcium salt and adjuvant poly(I:C) failed to do so.

We then examined the influence of AnCHNPs on DC migration in a transwell assay, where B16F10 cells, with or without preirradiation (100 Gy), were seeded in the lower chamber and CFSE-labeled BMDCs were loaded onto the inset (Figure 3e). Compared to unirradiated cells, those receiving preirradiation were associated with enhanced DC transwell migration (Figure 3e). This is due to radiation-induced release of chemokines that promote chemotactic movement, which has been observed by others.^38^ Incubation with AnCHNPs further increased the number of DCs migrating to the lower chamber, suggesting the ability of the nanoparticles to enhance the ability of DCs to sense chemotactic signals and move toward the source. In comparison, calcium salt had a minimal effect on DC migration (Figure 3e).

**Figure 4:**
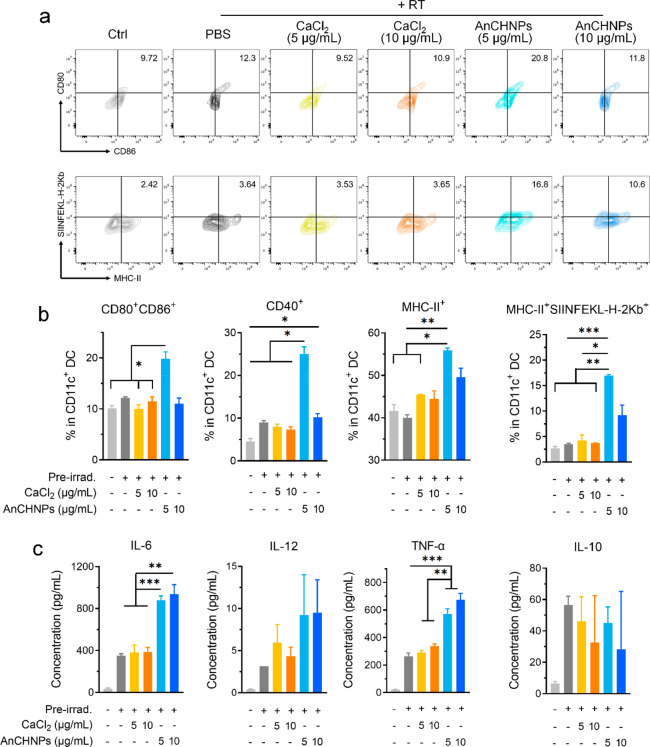
Impact of AnCHNPs on DC maturation, tested *in vitro* with a coculture of BMDCs and B16F10-OVA (preirradiated, 100 Gy) in the presence of AnCHNPs or CaCl_2_ (5 or 10 μg/mL) (*n* = 3). (a) Quadrant graphs showing the changes of CD80^+^CD86^+^ and MHC-II^+^SIINFEKL-H-2K^b+^ populations among DCs, analyzed by flow cytometry. In the control group, live B16F10-OVA cells were used in the coculture, and PBS was added into the incubation medium. (b) Bar graphs showing the frequencies of CD80^+^CD86^+^, CD40^+^, MHC-II^+^, and MHC-II^+^SIINFEKL-H-2K^b+^ cells among DCs (*n* = 3). (c) Pro- (IL-6, IL-12, and TNF-α) and anti-inflammatory (IL-10) cytokines in the supernatant of the coculture, analyzed by ELISA (*n* = 3). Data are presented as the mean ± s.d. *, *p* < 0.05; **, *p* < 0.01; ***, *p* < 0.001.

Next, we examined maturation and activation of DCs when they were cocultured with preirradiated (100 Gy) B16F10-OVA cells. Incubation with AnCHNPs significantly increased the frequency of CD80^+^CD86^+^ DCs in this setting (Figure 4a). Other DC maturation markers, including CD40 and MHC-II, were also upregulated upon incubation with AnCHNPs (Figure 4b). In addition, there was a significant increase in the surface SIINFEKL-H-2K^b^, indicating enhanced antigen presentation (Figure 4b). In comparison, calcium salt and silica nanoparticles did not positively affect DC activation (Figure S4). We also measured cytokines in the supernatant of the cocultures. Compared to DCs treated with calcium salt or carrier alone, those treated with AnCHNPs showed increased secretion of pro-inflammatory cytokines, including IL-6, IL-12, and TNF-α (Figure 4c), but decreased secretion of IL-10 (although not significant, *p* = 0.3307). Notably, AnCHNPs were more efficient at 5 μg/mL than at 10 μg/mL, which was likely due to a negative effect of the nanoparticles on cell viability at the higher concentration (Figure S3).

Taken together, these *in vitro* results support the idea that AnCHNPs effectively enhance the maturation, migration, and antigen presentation of DCs.

### Mechanisms Underlying DC Activation by AnCHNPs

**Figure 5:**
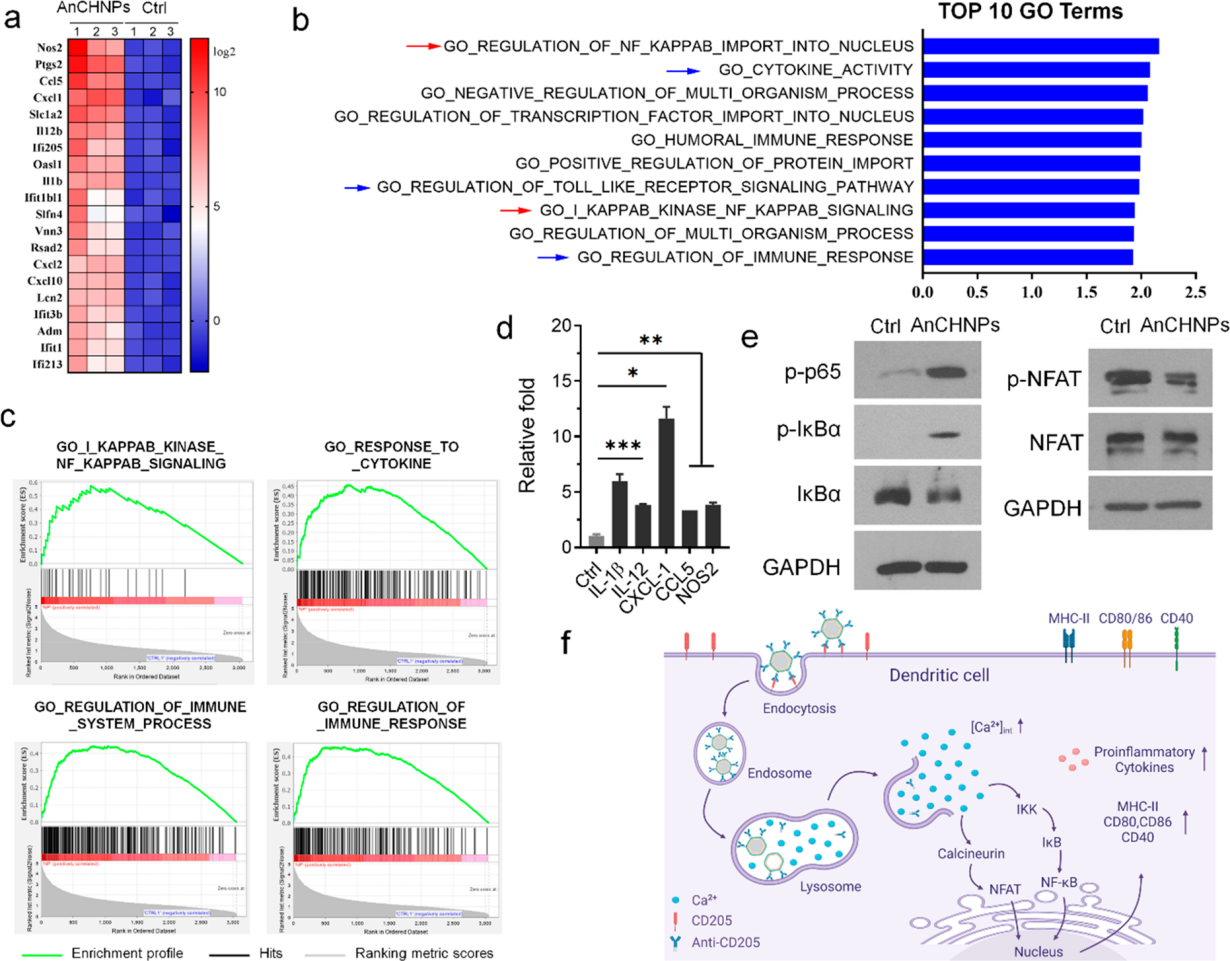
Understanding the mechanism behind DC activation by AnCHNPs. (a) A heatmap showing the top 10 most upregulated genes in AnCHNPs-treated BMDCs (vs Ctrl) (*n* = 3). (b) GO enrichment analysis of the top 10 GO terms resulting from upregulated DGEs in AnCHNPs-treated BMDCs (vs Ctrl). (c) GSEA analysis of enrichment plots for a priori gene sets for the top 4 most upregulated pathways in AnCHNPs-treated BMDCs (vs Ctrl). GSEA, gene set enrichment analysis; NES, normalized enrichment score. (d) Expression of selected cytokine and chemokine genes by RT-qPCR (*n* = 3). Data are presented as the mean ± s.d. *, *p* < 0.05; **, *p* < 0.01; ***, *p* < 0.001. (e) Western blot examining proteins of interest. BMDCs were treated with OVA (10 μg/mL) (Ctrl) or OVA (10 μg/mL) plus AnCHNPs (5 μg/mL) for 24 h. (f) Schematic illustration of the activation mechanism. The endocytosis of AnCHNPs is followed by particle degradation in the lysosome and Ca^2+^ release into the cytosol. The increase in [Ca^2+^]_int_ leads to the activation of the NF-κB and NFAT pathways, eliciting antigen-presenting molecules, costimulatory molecules, and pro-inflammatory cytokines.

To understand the changes in gene expression occurring in DC cells with or without AnCHNPs, we performed whole transcriptome sequencing. Differentially expressed genes (DEGs) analysis revealed that 1325 genes were upregulated (fold change >1.5 and *p* < 0.05) and 3049 genes were downregulated (fold change <0. Five and *p* < 0.05) in AnCHNP-treated mouse BMDCs. Interestingly, nitric oxide synthase 2 (Nos2), a reactive free radical that acts as a biological mediator in antitumor activity, was the most upregulated gene in BMDCs after the AnCHNP treatment (Figure 5a). Gene ontology (GO) enrichment analysis revealed that gene signatures of NF-κB signaling, cytokine activity, and immune response were among the top 10 most upregulated GO terms in AnCHNP-treated BMDCs compared to the control (Figure 5b). These results were consistent with the Gene Set Enrichment Analysis (GSEA) using the same RNA-seq data (Figure 5c). These observations were validated by qRT-PCR, which showed that treatment with AnCHNPs induced chemokines (e.g., CXCL-1, CCL5, CXCL2, and CXCL10) and cytokines (e.g., IL-1β, IL-12, and IL-6) (Figure 5d), which are known to attract and stimulate immune cells, including T cells. We also performed Western blotting to examine the activation pathways of the BMDCs (Figure 5e). Compared with controls, BMDCs treated with AnCHNPs showed increased phosphorylation of IκBα and p-65, indicating the activation of the NF-κB pathway. Meanwhile, AnCHNPs treatment also resulted in increased levels of dephosphorylation of NFAT, suggesting the activation of the NFAT axis (Figure 5e). These results are consistent with previous reports that the NF-κB and NFAT pathways are involved in calcium signaling.^27^

Overall, our results suggest that sustained release of calcium from AnCHNPs leads to activation of both the NF-κB and NFAT pathways, inducing chemokines, cytokines, antigen-presenting molecules, and costimulatory molecules, thereby enhancing DC-mediated immunity (Figure 5f).

### Effect of AnCHNPs on Immune Responses *in Vivo*

**Figure 6:**
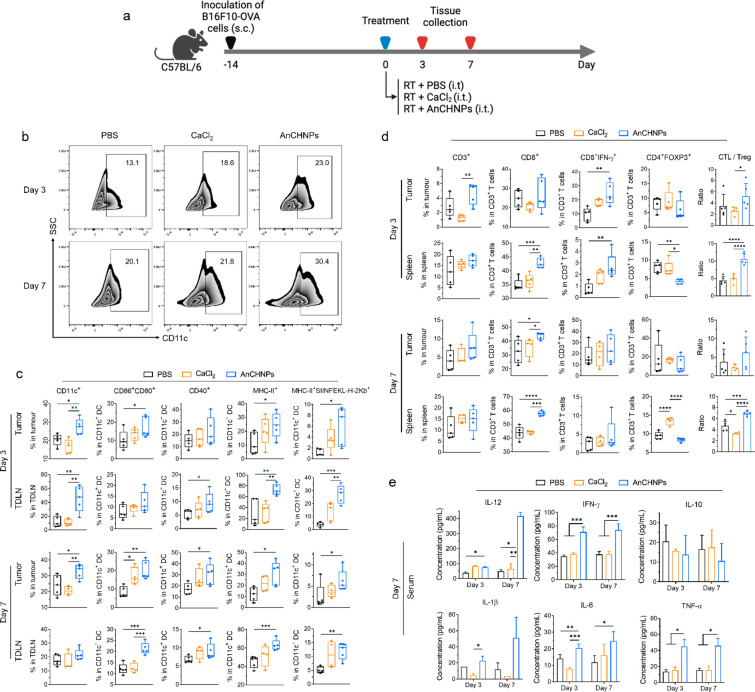
Impact of AnCHNPs on immune responses, tested *in vivo* in B16F10-OVA-tumor-bearing C57BL/6 mice. (a) Experimental scheme. On Day 0, the animals received radiation (10 Gy) applied to tumors, followed by i.t. administration of AnCHNPs (200 μg/kg) (RT + AnCHNPs; *n* = 10). CaCl_2_ plus RT (RT + CaCl_2_) and PBS plus RT (RT + PBS) were tested in control groups (*n* = 10). Half of the animals from each group were euthanized on Day 3, while the rest were euthanized on Day 7. Tumor, TDLNs, and spleen tissues were harvested for flow cytometry. Serum samples were collected for ELISA analysis. (b) Overall DC population in tumors on Day 3 and 7. (c) Populations of CD86^+^CD80^+^, CD40^+^, MHC-II^+^, and MHC-II^+^SIINFEKL-H-2K^b+^ DCs in tumors and TDLNs on Day 3 and Day 7. (d) T lymphocyte populations, including CTLs (CD45^+^CD3^+^CD8^+^), effector CTLs (IFN-γ^+^CD45^+^CD3^+^CD8^+^), and Tregs (CD45^+^CD3^+^CD4^+^Foxp3^+^), in both the tumor and spleen on Day 3 and Day 7. CTL/Treg ratios were also calculated. (e) Serum cytokine levels, including IL-12, IFN-γ, IL-10, IL-1β, IL-6, and TNF-α, on Day 3 and Day 7. Data are presented as the mean ± s.d. *, *p* < 0.05; **, *p* < 0.01; ***, *p* < 0.001; ****, *p* < 0.0001.

We then investigated the effect of AnCHNPs *in vivo*. We tested this in B16F10-OVA tumor-bearing C57BL/6 mice. Tumors were first irradiated (10 Gy), which was expected to induce the release of tumor-associated antigens and DAMPs. This was followed by intratumoral (i.t.) administration of AnCHNPs (200 μg/kg) after 1 h. For comparison, CaCl_2_ or vehicle alone (PBS) was injected i.t. Animals were euthanized on Day 3 or 7, and tumors, spleens, and tumor draining lymph nodes (TDLNs) were harvested for flow cytometric analysis (Figure 6a).

Compared to the PBS or CaCl_2_ controls, mice treated with AnCHNPs showed a significant increase in CD11c^+^ cells in the tumors on Day 3 and Day 7, indicating increased tumor infiltration of DCs (Figure 6b). The populations of MHC-II^+^ and CD80^+^CD86^+^ were significantly increased (Figure 6c), suggesting enhanced DC maturation. In addition, AnCHNPs caused an increase in SIINFEKL-H-2K^b+^ DCs in tumors on Day 3, indicating improved antigen presentation (Figure 6c). Similarly, we observed increased populations of MHC-II^+^, CD80^+^CD86^+^, CD40^+^, and SIINFEKL-H-2K^b+^ DCs in TDLNs on Day 3 (Figure 6c), which is attributed to the enhanced migration of DCs as a result of activation by AnCHNPs.

We also examined T lymphocytes in the tumors. AnCHNPs significantly promoted the tumor infiltration of cytotoxic T cells (CTLs, CD45^+^CD3^+^CD8^+^). The population of effector T cells (IFN-γ^+^ CTLs) was increased on Day 3 (Figure 6d). Meanwhile, the frequency of Tregs (CD45^+^CD3^+^CD4^+^Foxp3^+^) was reduced (though insignificant, Figure 6d). The tumor CTL/Treg ratio was increased by ∼2-fold in the AnCHNPs group on Day 3, indicating a strong boost of intratumoral immunity (Figure 6d). Similar trends were also observed for T lymphocytes in TDLNs (Figure S5) and spleen (Figure 6d). In comparison, CaCl_2_ had a minimal effect on either CTLs or Tregs in tumors.

Furthermore, we examined antigen-specific cellular immunity *ex vivo* using a coculture of splenocytes and B16F10-OVA cells. Compared to splenocytes from the PBS control group, those from the AnCHNPs group showed an increased IFN-γ^+^ CTL population throughout the incubation (Figure S6), supporting the notion that the nanoparticles elicited a systemic antitumor immunity. Conversely, splenocytes taken from the CaCl_2_ group showed marginal T cell activation in the coculture.

Finally, the serum from different treatment groups was analyzed for cytokine levels. Compared to the PBS control, animals treated with AnCHNPs, but not with CaCl_2_, showed elevated levels of IL-1β, IL-6, TNF-α, IFN-γ, and IL-12 but a decreased level of IL-10, on both Day 3 and Day 7 (Figure 6e), consistent with the results of leucocyte profiling.

Collectively, our results suggest that AnCHNPs can promote DC maturation and migration, which in turn can augment both innate and cellular immunity against tumors.

### Evaluation of the Efficacy of AnCHNPs When Used in Combination with Irradiation

**Figure 7:**
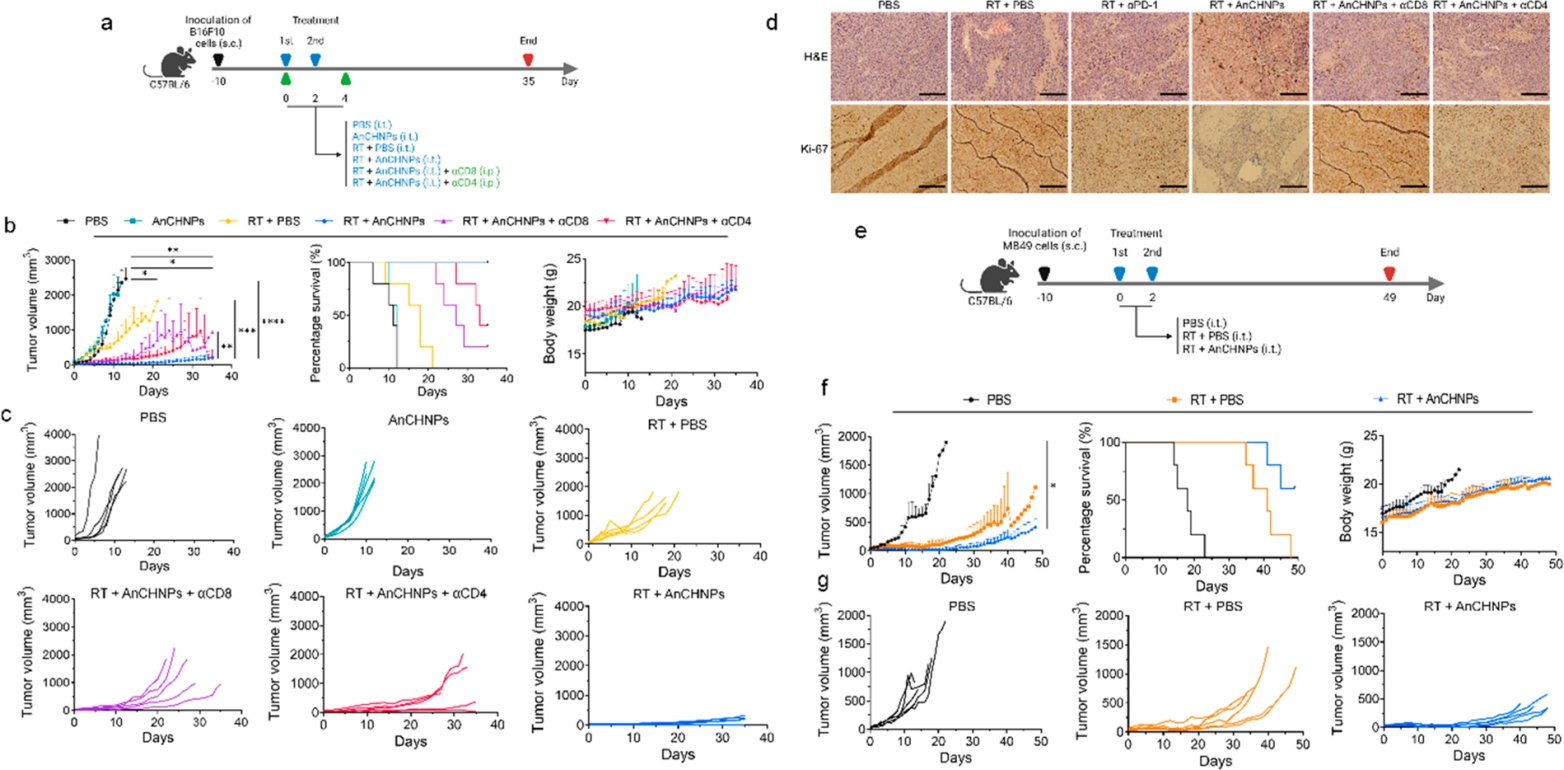
Therapeutic benefits of AnCHNPs when used in combination with RT, tested in both B16F10 and MB49 tumor bearing C57BL/6 mice. (a–d) Therapy study with the B16F10 model. (a) Scheme of the experiment. On Day 0 and Day 2, animals received radiation (10 Gy) applied to tumors, followed by i.t. administration of 200 μg/kg AnCHNPs (RT+AnCHNPs; *n* = 5). PBS alone (PBS), PBS plus RT (RT+PBS), and AnCHNPs alone (AnCHNPs) were also tested (*n* = 5). Moreover, anti-CD4 or anti-CD8 antibodies (10 mg/kg, injected i.p. on Day 0 and Day 4) were administered in addition to the RT-AnCHNPs combination (RT+AnCHNPs+αCD4 and RT+AnCHNPs+αCD8, respectively; *n* = 5). (b) Average tumor growth, animal survival, and body weight curves. (c) Individual tumor growth curves. (d) Post-mortem H&E and Ki67 staining of tumor tissues taken from different treatment groups. Scale bars, 200 μm. (e–g) Therapy study with the MB49 model. (e) Scheme of the MB49 experiment. On Day 0 and Day 2, animals received radiation (10 Gy) applied to tumors, followed by i.t. administration of AnCHNPs (200 μg/kg) (RT+AnCHNPs; *n* = 5). PBS alone (PBS) and PBS plus RT (RT+PBS) were also tested (*n* = 5). (f) Average tumor growth, animal survival, and body weight curves. (g) Individual tumor growth curves. Data are presented as the mean ± s.d. *, *p* < 0.05; **, *p* < 0.01; ***, *p* < 0.001; ****, *p* < 0.0001.

Next, we evaluated the therapeutic benefit of AnCHNPs when they are used in combination with other treatments, starting with RT. This was first tested in B16F10 tumor-bearing C57BL/6 mice. Specifically, AnCHNPs (50 μL, 200 μg/kg, in PBS) were injected i.t. 1 h after radiation (10 Gy) to the tumor, while the rest of the animal body was lead-shielded. A total of two treatments were performed 2 days apart (RT+AnCHNPs). For comparison, animals were treated with vehicle alone, RT alone, or AnCHNPs alone (Figure 7a).

Tumors in the PBS group grew rapidly, with all the animals either dying or reaching a humane end point by 2 weeks (Figure 7b). RT moderately inhibited tumor growth, but all animals in this group died within 3 weeks. In comparison, AnCHNPs with RT significantly improved tumor suppression. Eighty percent of the animals in the combination group experienced tumor regression within the first 3 weeks (Figure 7c). All animals in this group were alive at 5 weeks, and 20% of them remained tumor free. Notably, AnCHNPs alone had no effect on tumor growth (Figure 7b,c), suggesting that the therapeutic benefit is due to the immunomodulatory capabilities of the nanoparticles rather than their direct tumoricidal effects or influence on tumoral pH. This notion is supported by results from T cell depletion controls, where animals received either anti-CD4 or anti-CD8 antibodies in addition to the AnCHNPs-RT combination. Either CD4 or CD8 T cell depletion worsened the treatment outcomes (Figure 7b,c, Figure S7). Between the two groups, anti-CD8 antibodies were more effective in abrogating the therapeutic benefit (Figure 7b,c), suggesting enhanced cellular immunity as a major cause of the radiosensitization.

We performed post-mortem histopathology on tumor and major organ specimens. Hematoxylin/eosin (H&E) staining exhibited large areas of nuclear shrinkage and fragmentation in tumors treated with AnCHNPs plus radiation (Figure 7d). This was accompanied by a reduced level of positive *K*_i_-67 staining in the combination group, indicating a decreased level of cell proliferation. Meanwhile, no signs of toxicity were observed in any of the major organs (Figure S8).

To validate the efficacy, we also tested AnCHNPs with RT in MB49 tumor-bearing C57BL/6 mice (Figure 7e). RT alone was more effective in this model, extending the median survival from 17 days to 40 days. The addition of AnCHNPs to the regimen increased the efficacy. Compared to RT alone, tumor growth suppression was improved by 65.9% in the AnCHNPs-RT combination group at Day 40. At the end of the experiment at Week 7, 60 percent of the animals in the combination group were alive. In comparison, all animals in the PBS or RT group had died by this time (Figure 7f,g).

In conclusion, our *in vivo* studies show that AnCHNPs can enhance RT-induced immunity, resulting in improved tumor control and animal survival.

### Evaluation of the Efficacy of AnCHNPs When Used in Combination with Chemotherapy or Immunotherapy

**Figure 8:**
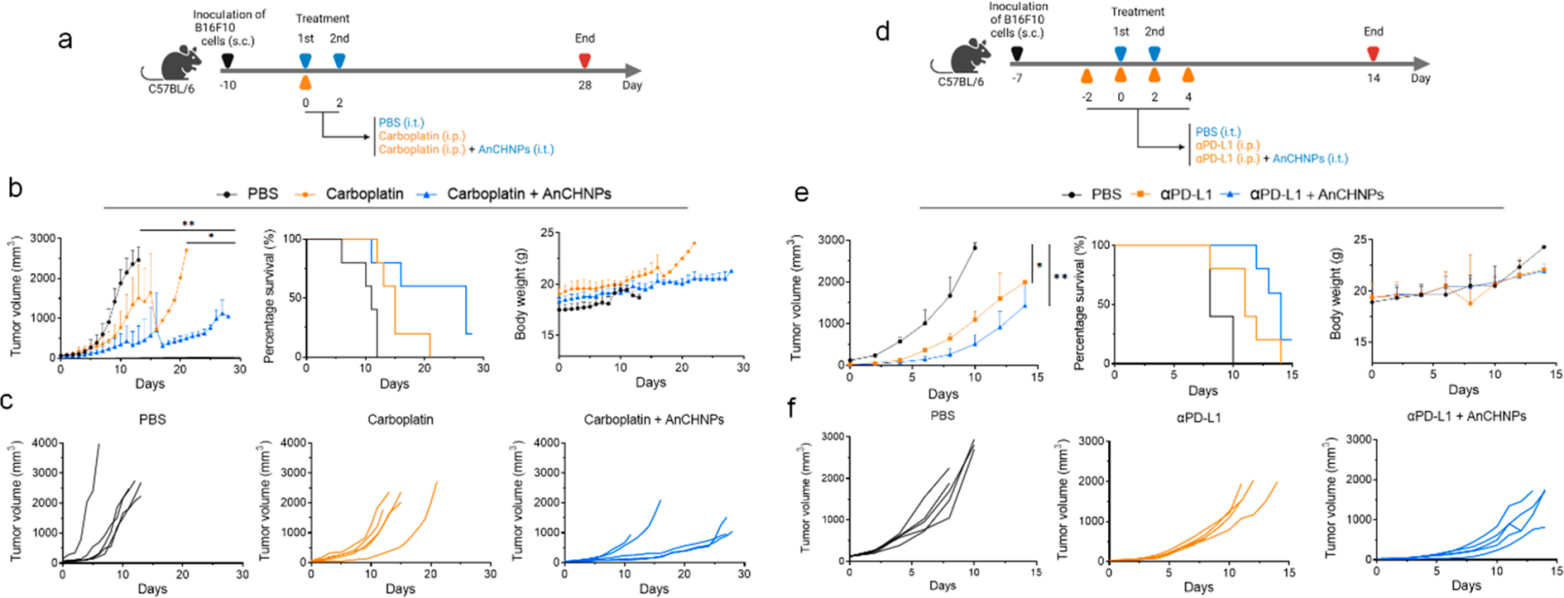
Therapeutic benefits of AnCHNPs when used in combination with chemotherapy or immunotherapy, tested in B16F10-tumor-bearing mice. (a–c) Dual therapy with AnCHNPs and carboplatin. (a) Scheme of experiment. On Day 0 and Day 2, animals received carboplatin (i.p., 40 mg/kg on Day 0), followed by i.t. administration of 200 μg/kg AnCHNPs (carboplatin+AnCHNPs; *n* = 5). PBS alone (PBS) and carboplatin alone (carboplatin) were tested for comparison (*n* = 5). (b) Average tumor growth, animal survival, and body weight curves. *, *p* < 0.05; **, *p* < 0.01. (c) Individual tumor growth curves. (d–f) Dual therapy with AnCHNPs and anti-PD-L1 antibodies, tested in B16F10-tumor-bearing mice. (d) Scheme of the experiment. On Day −2, 0, 2 and 4, animals received anti-PD-L1 antibodies (i.p., 10 mg/kg), followed by i.t. administration of 200 μg/kg AnCHNPs (αPD-L1+AnCHNPs; *n* = 5). PBS alone (PBS) and anti-PD-L1 alone (αPD-L1) were tested for comparison (*n* = 5). (e) Average tumor growth, animal survival, and body weight curves. *, *p* < 0.05; **, *p* < 0.01. (f) Individual tumor growth curves.

We next investigated whether AnCHNPs could enhance the efficacy of chemotherapy such as carboplatin. We first tested this in B16F10 tumor models using a combination of carboplatin (40 mg/kg, ip) and AnCHNPs (200 μg/kg, i.t.) (Figure 8a). Carboplatin is a well-known ICD agent, but as a monotherapy it is inefficient to induce a robust immunity.^39^ Carboplatin alone only marginally delayed tumor growth (Figure 8b,c), and all animals in this group died within 3 weeks. The addition of AnCHNPs significantly improved the treatment outcomes (Figure 8b,c), extending median survival from 15 days in the carboplatin group to 23 days in the combination group. No additional toxicities were observed (Figure S8).

We also investigated whether AnCHNPs could enhance the efficacy of an immune checkpoint blockade (Figure 8d). Specifically, we systemically administered anti-PD-L1 antibody (i.p., 10 mg/kg, 4 doses) to B16F10 tumor-bearing mice and i.t. injected AnCHNPs (200 μg/kg) on Day 0 and Day 2. Compared to αPD-L1 alone, AnCHNPs improved tumor suppression (Figure 8e,f). The combination was again well tolerated by the animals (Figure S9).

Collectively, our results suggest that AnCHNPs can enhance the efficacy of chemotherapy and immunotherapy without causing additional toxicity.

## Conclusions

In this study, we investigated AnCHNPs as an immunomodulatory agent. AnCHNPs enter DCs by endocytosis and are degraded in lysosomes, releasing calcium into the cytosol. Normally, DCs are activated by sensing external stimuli such as pathogens or damaged tissue through pattern recognition receptors. This would trigger a cascade of events leading to the depletion of the calcium store, activation of the Ca^2+^ release-activated Ca^2+^ channels, and then an influx of calcium.^22^ In contrast, AnCHNPs deliver calcium directly into the cytosol. By bypassing the upstream signaling, this approach activates the NFAT and NF-κB pathways and stimulates DCs even in an immunosuppressive environment (Figure 3d).

While calcium nanoparticles have been used in applications such as gene or drug delivery,^30,31^ the effect of calcium nanoparticles on immune cells, particularly DCs, has rarely been investigated. Several recent studies have shown that calcium nanoparticles can enhance the immune response,^32,40−42^ but the focus has been on the effect of the calcium nanoparticles on the extracellular pH^32^ or on disrupting autophagy inhibition^32^ rather than on the intracellular calcium levels. Note that calcium released into the extracellular environment contributes little to [Ca^2+^]_int_ (Figure 2f) and does not alter DC function (Figures 3 and 4). It is worth noting that previous studies find that bare calcium nanoparticles do not significantly affect [Ca^2+^]_int_, even when taken up by cells,^43^ which is attributed to rapid particle dissolution and calcium efflux. In contrast, we show that calcium nanoparticles can persistently increase calcium levels in DCs, which is key to their activation.^29^ The silica coating, which allows controlled calcium release, and the surface-bound anti-CD205 antibody, which allows receptor-mediated endocytosis by DCs, both contribute to effective immunomodulation.

A major advantage of calcium nanoparticles over conventional adjuvants is their high biocompatibility. As shown in our studies, the calcium core of AnCHNPs is largely degraded after 24 h. The resulting calcium ions, which cannot freely cross the plasma membrane, are safely excreted. In our study, AnCHNPs were injected at 200 μg/kg or ∼4 μg per mouse. This calcium dose was much lower than that used by others in attempts to increase the pH of the TME.^44^ Nanoparticles at such a low dose do not have tumoricidal effects by themselves (Figure 7b,c). Few of the i.t. administered nanoparticles entered the blood circulation and ended up in major organs (Figure S10). Complete blood count (Table S1) and serum biochemistry (Figure S11a,b) analyses found no abnormalities in mice injected with AnCHNPs. No significant increase in serum calcium level was observed (Figure S11c). Histopathology also revealed no signs of side effects to major organs (Figures S8 and 9). Moreover, flow cytometry analysis found that the injection caused minimal harm to DCs or tumor infiltrating immune cells (Figure S12). These data support the good biocompatibility of the nanoparticles at the test dose.

Meanwhile, AnCHNPs alone do not appear to be sufficient to overcome the immunosuppressive TME or improve therapeutic outcomes (Figure 7b). It is possible that the immunostimulatory effects are most pronounced when the nanoparticles are used in combination with treatments that can enhance tumor-associated antigen release and DC infiltration (e.g., RT and chemotherapy). Future studies are needed to investigate the effects of AnCHNP dose and injection timing to optimize the immunostimulatory effects. While nanoparticles with sizes similar to ours have been routinely tested as intratumoral formulations,^45−48^ we may explore calcium nanoparticles of other sizes, which may affect the intratumoral diffusion, uptake, and degradation of the nanoparticles, in turn influencing the immune response. It will also be interesting to load the calcium nanoparticles with tumor antigens to create DC-targeted vaccines. Overall, our investigation revisits an “old” nanomaterial but introduces a fresh perspective that could lead to the development of safe and effective immunomodulation strategies and cancer therapies.

## Methods

### Synthesis of Calcium Hydroxide or Ca(OH)_2_ Nanoparticles (CHNPs)

In a typical synthesis, 443.92 mg of calcium chloride (CaCl_2_, anhydrous, 97%, Sigma-Aldrich, Lot # SLBQ3073 V) was first dissolved in 18.571 mL of Milli Q H_2_O. Into the solution, 1.429 mL of 6 M sodium hydroxide (NaOH, Fisher, Lot # 166374) was dropwise added. The resulting solution was stirred magnetically at 90 °C for 5 min. The raw products were collected by centrifugation and then redispersed in ethanol (200 proof, Koptec, lot 274014) with brief sonication. The washing step was repeated 3 times to remove unreacted precursors.

### Synthesis of Silica-Coated Calcium Hydroxide Nanoparticles (SCHNPs)

Fifty milligrams of CHNPs were dispersed in a mixture solvent containing 40 mL of ethanol and 0.4 mL of ammonia (28.0–30.0%, J.T.Baker, Lot # 0000010971). The solution underwent vigorous stirring for 30 min. After sonication for 30 s, 300 μL of TEOS (tetraethyl orthosilicate, 98%, Sigma-Aldrich, Lot # STBJ8253) was dropwise added into the solution, followed by the addition of 180 μL of APTES ((3-aminopropyl)triethoxysilane, 98%, Sigma-Aldrich, Lot # MKCM7627). The resulting solution underwent stirring at room temperature for 20 h. SCHNPs were collected by centrifugation and washed three times with ethanol.

### Synthesis of PEG-Diacid-Coated Calcium Hydroxide Nanoparticles (PCHNPs)

Twenty mg of SCHNPs were dispersed in 10 mL of DMSO (dimethyl sulfoxide, 99.9%, Sigma-Aldrich, Lot # MKBF8194 V) and transferred to a 20 mL glass vial. Under magnetic stirring, 200 mg PEG-diacid (M.W. 2,000, JenKem Tech, Lot # ZZ192P158), 20 mg EDC (*N*-(3-(dimethylamino)propyl)-*N*′-ethylcarbodiimide, 97%, Sigma-Aldrich, Lot # 507429), and 15 mg NHS (*N*-hydroxysuccinimide, 98%, Sigma-Aldrich, Lot # 130672), dissolved in 10 mL DMSO, were added into the nanoparticle suspension. The resulting solution underwent magnetic stirring at 60 °C for 20 h. PCHNPs were collected by centrifugation and washed 2 times with Milli Q H_2_O.

### Synthesis of Anti-CD205-Antibody-Conjugated Calcium Hydroxide Nanoparticles (AnCHNPs)

PCHNPs (0.5 mg) were dispersed in 1 mL of cold sterile PBS and kept under magnetic stirring at 4 °C. Ten μL of anti-CD205 antibodies (mouse monoclonal HD30, Sigma-Aldrich, Lot # 531834) was added into the PCHNP solution. After 25 min, 2 μL of ethanolamine (99%, Sigma-Aldrich, Lot # 398136) was added into the solution. After reaction for another 5 min, AnCHNPs were collected by centrifugation and washed with PBS once. The total amount of antibody conjugated was measured by protein assay, and the amount of calcium was measured by ICP-MS. The number of antibody molecules bound per nanoparticle was calculated by dividing the number of protein molecules by the estimated number of nanoparticles, assuming hexanol plates with a density of 2.24 g/cm^3^. Fresh-made AnCHNPs were used for subsequent *in vitro* and *in vivo* studies, unless specified otherwise. All nanoparticle doses were expressed in Ca concentrations unless specified otherwise.

### Physiochemical Characterizations of Nanoparticles

Scanning electron microscopy (SEM) and energy dispersive X-ray spectroscopy (EDS) elemental mapping images were acquired on an FEI Teneo field emission SEM equipped with an Oxford EDS system. Transmission electron microscopy (TEM) was carried out on an FEI Tecnai20 transmission electron microscope operating at an accelerating voltage of 200 kV. High resolution TEM was performed on a Hitachi transmission electron microscope H9500 operating at a 300 kV accelerating voltage. X-ray diffraction (XRD) analysis was carried out on a Bruker D8-Advance system using dried samples placed on a cut glass slide with Cu Kα1 radiation (λ = 1.5406 Å). Dynamic light scattering (DLS) and zeta potential measurements were carried out on a Malvern Zetasizer Nano ZS system. Fourier-transform infrared (FT-IR) spectra were recorded on a Nicolet iS10 FT-IR spectrometer.

### Nanoparticle Stability and Calcium Release

CHNPs and PCHNPs were dispersed in 100 μL of ammonium acetate buffer solutions (pH = 5.5 or 7.4) and loaded onto a Slide-A-LyzerTM MINI Dialysis Device (MWCO: 2K, cat. no. 69550, ThermoFisher, US). The dialysis unit was put into a 5 mL Eppendorf tube containing 4.5 mL of the same ammonium acetate buffer. The tube was placed on a shaker (20 rpm) at room temperature. At different time points (0, 0.25, 0.5, 1, 2, 4, 8, 10, and 24 h), 500 μL solution was taken from the Eppendorf tube, and its Ca^2+^ content was measured by a calcium ion-selective electrode (HORIBA LAQUAtwin Ca-11). A 500 μL aliquot of fresh buffer was added back into the Eppendorf tube to keep the total volume at 4.5 mL. All samples were analyzed in triplicates. In addition, TEM images were acquired for PCHNP samples taken at 0, 2, 4, 8, 12, and 24 h.

### Cell Culture

B16F10-OVA cells (murine melanoma) were grown in high glucose DMEM (ATCC 30–2002TM) supplemented with the G418 ingredient. B16F10 cells (murine melanoma) were grown in high glucose DMEM (ATCC 30–2002TM). Bone marrow derived dendritic cells (BMDCs) were established from germ cells extracted from the bone marrow of C57BL/6 mice and cultured in RPMI-1640 (Corning, 10-040-CV) containing GM-SCF according to a published protocol.^49^ MB49 cells (murine bladder carcinoma) were grown in RPMI-1640 (Corning, 10-040-CV). All cell culturing media were supplemented with 10% fetal bovine serum (FBS), 100 units/mL penicillin, and 100 units/mL streptomycin (MediaTech, USA). All cells were maintained in a humidified 5% carbon dioxide atmosphere at 37 °C.

### Cell Cytotoxicity

ATPlite-1step luminescence assay kit (PerkinElmer, Lot # 107–21051) was used to determine cellular ATP contents following the manufacturer’s protocol. BMDCs were seeded into 96-well plates at a density of 1 × 10^4^ cells per well and incubated overnight. The cells were then treated with CaCl_2_ solution, AnCHNPs, and PEGylated SiO_2_ nanoparticles without the calcium core (by preaging) at a dose ranging from 0.05 to 100 μg/mL for 24 h. The luminescence intensity of each well was measured on a microplate reader (Synergy Mx, BioTeK) and normalized to control cells.

### Cell Uptake

BMDCs were seeded into 6-well plates at a density of 1 × 10^6^ cells per well and incubated overnight. The cells were then treated with Cy5-labeled PCHNPs and AnCHNPs (5 μg/mL) for 2 h. Furthermore, endocytosis inhibitors including sodium azide (NaN_3_, 99.5%, Sigma-Aldrich, Lot # S2002), dynasore (C_18_H_14_N_2_O_4_, 98%, Sigma-Aldrich, Lot # 324410), nystatin (Sigma-Aldrich, Lot # N4014), and chlorpromazine (C_17_H_19_ClN_2_S·HCl, 98%, Sigma-Aldrich, Lot # C8138) were coapplied. The fluorescence of Cy5 in DCs was measured by flow cytometry.

### Lysosomal pH

LysoSensor Yellow/Blue DND-160 (PDMPO) kit (Invitrogen, Lot # 2174576) was used to measure the lysosomal pH of BMDCs taking up AnCHNPs. Briefly, BMDCs were seeded into a 96-well plate at a density of 1 × 10^4^ cells per well and incubated overnight. At different time points (0, 1, 2, 4, 8, and 24 h), incubation medium was removed and replenished with prewarmed (37 °C) medium containing the probe (1 μM). After incubation for 5 min, the medium was replaced with fresh culturing medium, and the fluorescence (dual excitation at 329 and 384 nm, and emissions at both 440 and 540 nm) was measured on a microplate reader (Synergy Mx, BioTeK). The lysosomal pH was estimated based on the blue/yellow fluorescence ratio according to the vendor’s protocol.

### Measurement of [Ca^2+^]_int_

Fluo-3 AM (Cayman, 14960) was used to measure [Ca^2+^]_int_ in BMDCs after treatment with the AnCHNPs. Briefly, BMDCs were seeded into a 96-well plate at a density of 1 × 10^4^ cells per well and incubated overnight. After cells were incubated with AnCHNPs for different times (0, 1, 2, 4, 8, and 24 h), the medium was removed and replenished with prewarmed (37 °C) fresh medium containing the probe (to a final concentration of 5 μM). After 30 min, the medium was aspirated and replaced with fresh medium. Cells were incubated for another 30 min to allow for complete de-esterification of the acetoxymethyl esters. Fluorescence (ex/em: 485/520 nm) was recorded on a microplate reader (Synergy Mx, BioTeK).

### [Na^+^]_int_ and [K^+^]_int_ Measurement

SBFI-AM (sodium-binding benzofuran isophthalate acetoxymethyl ester, Setareh Biotech, Lot # 50609), and PBFI-AM (potassium-binding benzofuran isophthalate acetoxymethyl ester, Setareh Biotech, Lot # 5027) were used to measure [Na^+^]_int_ and [K^+^]_int_ in BMDCs, respectively, by following the vendor’s protocols. Briefly, BMDCs were seeded into a 96-well plate at a density of 1 × 10^4^ cells per well and incubated overnight. After cells were incubated with AnCHNPs for different times (0, 1, 2, 4, 8, and 24 h), the medium was removed and replenished with prewarmed (37 °C) fresh medium containing either SBFI-AM or PBFI-AM (final concentration of 10 μM). Cells were incubated for 30 min under the same growth conditions. Then the loading solution was replaced with fresh medium, removing dye molecules nonspecifically attached to the cell surface. Fluorescence (ex: 340/380 nm, em: 505 nm) was recorded on a microplate reader (Synergy Mx, BioTeK), and the ratio was used to determine the concentrations of Na^+^ and K^+^, respectively.

### Investigate BMDCs’ Maturation, Migration, and Antigen Presentation *in Vitro*

#### Maturation

BMDCs were seeded onto a 6-well plate at a density of 1 × 10^6^ cells per well 1 day before the experiment. BMDCs were treated with PBS, a CaCl_2_ solution (5 or 10 μg/mL), and AnCHNPs (5 or 10 μg/mL). After incubation for 24 h, the supernatant was removed, and BMDCs were harvested by a cell lifter. BMDCs were subsequently stained with MHCII-FITC (#107616) and CD205-APC (#138206) and analyzed by flow cytometry. In addition, BMDCs were treated with PEGylated SiO_2_ shell (10 μg/mL) for 24 h and then stained with MHCII-FITC (#107616), CD80-PerCP-Cy5.5 (#560526), CD86-BV605 (#563055), CD40-PE (#12-0401-83), and OVA-APC (#17-5743-82) before flow cytometry.

#### Migration

B16F10-OVA cells (preirradiated, 100 Gy) were seeded into the lower chamber of a 6-well Transwell Permeable Support system at a density of 1 × 10^5^ cells per well. For comparison, unirradiated B16F10-OVA cells were used. CFSE-labeled BMDCs at a density of 1 × 10^6^ cells per well were seeded to the upper chamber of the well. BMDCs were treated with PBS, CaCl_2_ (5 or 10 μg/mL), or AnCHNPs (5 or 10 μg/mL). LPS (1 μg/mL) was tested as a positive control. After 24 h incubation, cells in the lower chamber were harvested by a cell lifter and readied for flow cytometry. Percentages of CFSE positive cells were quantified.

#### Activation and Antigen Presentation

B16F10-OVA cells (preirradiated, 100 Gy) were seeded into a 6-well plate at a density of 1 × 10^5^ cells per well. For comparison, unirradiated B16F10-OVA cancer cells were tested. BMDCs at a density of 1 × 10^6^ cells per well were seeded into each well. The cocultures were treated with PBS, CaCl_2_ solution (5 or 10 μg/mL), or AnCHNPs (5 or 10 μg/mL). After 24 h incubation, the cells were harvested by a cell lifter, stained with MHCII-FITC (#107616), CD80-PerCP-Cy5.5 (#560526), CD86-BV605 (#563055), CD40-PE (#12-0401-83), and OVA-APC (#17-5743-82) and analyzed by flow cytometry. In addition, the supernatant was collected, and its IL-6, IL-10, IL-12, and TNF-α contents were measured by ELISA using R&D Systems Mouse IL-6, IL-10, IL-12, and TNF-α DuoSet kits (Minneapolis, MN). The results were analyzed using the Four Parameter Logistic Curve method by Myassay.com.

### RNA Sequencing (RNA-seq) and Data Analysis

BMDCs were seeded onto a 100 mm Petri dish at a density of 1 × 10^6^ cells per well and incubated overnight. Cells were treated with OVA (10 μg/mL) or OVA (10 μg/mL) plus AnCHNPs (5 μg/mL). After incubation for 12 h, cells were harvested by a cell lifter. The NucleoSpin RNA kit (Takara, Lot # 2010/002) was used for extracting RNA from three independent samples of BMDCs with different treatments. RNA quality was analyzed using a 2100 Bioanalyzer (Agilent Technologies, Santa Clara, CA). The purified RNA samples were sent to Novogene Corporation (Sacramento, CA) for library construction and sequencing using the Illumina HiSeq 2000 platform to obtain expression libraries of 50-nt read length. RNaseq data were analyzed as previously described.^50^ In brief, differentially expressed genes (DEGs) were identified using the DESeq R package functions estimateSizeFactors and nbinomTest. The *P* value <0.05, and fold change >1.5 or fold change <0. Five was set as the threshold for significantly differential expression. Hierarchical cluster analysis of DEGs was performed to explore transcript expression patterns, and Gene Ontology (GO) was performed to identify the potential function of all DEGs. GSEA was conducted using GSEA desktop application software with annotated gene sets of Molecular Signature Database v6.2. The detailed RNA-seq information on this assay is available in GSE208276 deposited in the NIH Gene Expression Omnibus (GEO) database.

### qRT-PCR

qRT-PCR was performed on a QuantStudio 3 system, using SYBR Green as an indicator. The PCR reaction mixture included 10 ng of cDNA, 500 nM of each primer (synthesized by Sigma, St. Louis, MO), 5 μL of 2× SYBR Green PCR Master Mix (Quantabio, cat. no. 101414–284), and RNase-free water which was added to increase the final volume to 10 μL. The qPCR reaction was carried out for 40 cycles at 95 °C for 15 s and 60 °C for 1 min. The data were quantified based on the ^ΔΔ^Ct method using GAPDH and histone as internal standards for normalization. Melting curve analysis for all qRT-PCR products was performed, which showed a single DNA duplex. Primer sequences are

- NOS2: For 5′-AGAGCCACAGTCCTCTTTGC-3′; Rev 5′-GCTCCTCTTCCAAGGTGCTT-3′.
- CCL5: For 5′-CTGCTGCTTTGCCTACCTCT-3′; Rev 5′-CGAGTGACAAACACGACTGC-3′.
- CXCL1: For 5′-CTGGGATTCACCTCAAGAACATC-3′; Rev 5′-CAGGGTCAGGCAAGCCT C-3′.
- IL-12b: For 5′-ATGAGAACTACAGCACCAGCTTC-3′; Rev 5-ACTTGAGGGAGAAGTAGG AATGG-3′.
- IL-1b: For 5′-TCGTGCTGTCGGACCCATAT-3′; Rev 5′-GTCGTTGCTTGGTTCTCCTTGT-3′.

### Western Blot

BMDCs were seeded onto a 100 mm Petri dish at a density of 1 × 10^6^ cells per well and incubated overnight. The cells were then treated with OVA (10 μg/mL) or OVA (10 μg/mL) plus AnCHNPs (5 μg/mL). After incubation for 24 h, cells were harvested and lysed with a RIPA buffer supplemented with 1× proteinase inhibitor cocktail (Amresco). Protein concentration was determined using a bicinchoninic acid (BCA) protein assay (Thermo Fisher Scientific). Protein lysates were loaded onto 10% SDS-PAGE and transferred to a PVDF membrane. Nonspecific binding to the membrane was blocked by incubation with 5% nonfat milk at room temperature for 1 h. The membrane was incubated with primary antibodies at 4 °C overnight at dilutions specified by the manufacturer. This is followed by incubation with secondary antibodies for 1 h at room temperature and then treatment with ECL reagents (Thermo Fisher Scientific). The membrane was then exposed to X-ray films (Santa Cruz). All the imaging results were analyzed by ImageJ. The antibodies used are NFAT1 (Cell Signaling Cat # 4389S); phospho-IκBα, phospho-NF-κB p65 (Cell Signaling Cat # 9936T); and GAPDH (Cell Signaling Cat # 5174S).

### Animal Models

All experimental procedures were conducted following protocols approved by the Institutional Animal Care and Use Committee (IACUC) of the University of Georgia. C57BL/6 mice (female, 4 weeks old) were purchased from Envigo Laboratories and maintained under pathogen-free conditions. The animal models were established by subcutaneously injecting 2 × 10^5^ B16F10-OVA, B16F10, or MB49 cells in 50 μL PBS into the right hind limb of each mouse after 2 weeks of settlement (6 weeks old).

### Flow Cytometry to Profile Immune Cells

C57BL/6 mice bearing B16F10-OVA tumors were randomly divided into three groups (*n* = 10 for each group), which were treated with (1) 10 Gy X-ray irradiation (320kV) + PBS (50 μL), (2) 10 Gy X-ray irradiation + CaCl_2_ solution (200 μg/kg, i.t.), or (3) 10 Gy X-ray irradiation + AnCHNPs (200 μg/kg, i.t.). The treatment began when the tumor size reached ∼100 mm^3^ (Day 0). All injections were performed at five sites of the tumor to ensure good coverage. CaCl_2_ and AnCHNPs were injected in 50 μL of PBS, 1 h after the radiation. On Day 3, 5 mice from each group were euthanized. The rest of the animals were euthanized on Day 7. The tumor, spleen, and tumor-draining lymph node were harvested for immune response profiling. Tumors were cut into small pieces with scissors and digested by incubating in DMEM containing 1 mg/mL of collagenase type V (Worthington Biochemical Corporation) at 37 °C for 45 min. The digested tissues were gently meshed though a 250 μm cell strainer (Thermo scientific, Lot # UB2685874A). Red blood cells were lysed with Ack lysing buffer (Gibco) according to the manufacturer’s instructions. The single-cell suspensions were washed with cold sterile PBS and resuspended in the staining buffer. Following counting and aliquoting, cells were stained with fluorophore-conjugated antibodies for 30 min at 4 °C. The spleen and lymph node were processed following similar procedures, except that a 70 μm cell strainer (Corning Falcon, ref # 352235) was used and that no collagenase type V was used. The following antimouse antibodies from BD Biosciences were used: CD45-APC-Cy7 (#557659), CD4-BV605 (#563151), FoxP3-PE (#563101), CD11c-PE-Cy7 (#558079), CD86-BV605 (#563055), and CD80-PerCP-Cy5.5 (#560526). CD40-PE (#12-0401-83) was purchased from Invitrogen. OVA-APC (#17-5743-82) was purchased from eBioscience. MHCII-FITC (#107616), CD205-APC (#138206), IFN-γ-APC (#505810), CD3-FITC (#100206), and CD8-BV510 (#100752) were purchased from BioLegend. The live/dead assay kit was purchased from Thermal Fisher. Multiparameter staining was used to identify cell populations of interest, including cytotoxic T cells (CD45^+^CD3^+^CD8^+^), effector T cells (CD45^+^CD3^+^CD8^+^IFNγ^+^), Tregs (CD45^+^CD3^+^CD4^+^FoxP3^+^), DCs (CD11c^+^), and OVA^+^ DCs (CD11c^+^MHC-II^+^SIINFEKL-H-2K^b+^). For intracellular FoxP3 and IFN-γ staining, cells were fixed and permeabilized using the Permeabilization Solution Kit (BD, 554714) and washed before flow cytometry (Quanteon, Agilent). To assess tumor-specific T-cell response, splenocytes from different treatment groups were cocultured with B16F10-OVA cells for 6 h before staining and flow cytometry. The data were processed by FlowJo 10.0. Doublets were excluded based on forward and side scatter. Dead cells were excluded based on positive DAPI staining. In addition, blood samples were collected on Day 3 and 7 for cytokine analysis. Specifically, IL-1β, IL-6, IL-10, IL-12, TNF-α, and IFN-γ in the serum were measured using R&D Systems Mouse DuoSet ELISA kits (Minneapolis, MN) following the manufacturer’s protocol. Results were analyzed using the Four Parameter Logistic Curve method from Myassay.com.

### Therapy Studies

#### Combination with Radiotherapy

The experiments were performed in C57BL/6 mice bearing B16F10 or MB49 tumors. For B16F10 tumor models, when tumor sizes reached ∼50 mm^3^, the animals were randomized to receive the following treatments (*n* = 5 for each treatment group): (1) PBS (i.t., 50 μL × 2, Day 0 and Day 2), no irradiation; (2) AnCHNPs (i.t., 200 μg/kg × 2, Day 0 and Day 2); (3) RT (10 Gy × 2, Day 0 and Day 2) + PBS (i.t., 50 μL × 2, Day 0 and Day 2); (4) RT (10 Gy × 2, Day 0 and Day 2) + AnCHNPs (i.t., 200 μg/kg × 2, Day 0 and Day 2); (5) RT (10 Gy × 2, Day 0 and Day 2) + AnCHNPs (i.t., 200 μg/kg × 2, Day 0 and Day 2) + anti-CD8 antibodies (i.p., 10 mg/kg × 2, Day 0 and Day 4); (6) RT (10 Gy × 2, Day 0 and Day 2) + AnCHNPs (i.t., 200 μg/kg × 2, Day 0 and Day 4). All i.t. injections were performed at five sites of a tumor to ensure good coverage. Antibodies and AnCHNPs were injected in 100 and 50 μL PBS, respectively. AnCHNPs were injected 1 h after radiation if RT was applied. The tumor size and body weight were inspected daily. Tumors were measured in two dimensions with a caliper, and their volumes were calculated using (length) × (width)^2^/2. After therapy, tumors and major organs were collected and sectioned into 4-μm-thick slices for H&E and *K*_i_-67 staining. For MB49 tumor models, animals received the following treatments (*n* = 5 in each group): (1) PBS (i.t., 50 μL × 2, Day 0 and Day 2), no irradiation; (2) RT (10 Gy × 2, Day 0 and Day 2) + PBS (i.t., 50 μL × 2, Day 0 and Day 2); (3) RT (10 Gy × 2, Day 0 and Day 2) + AnCHNPs (i.t., 200 μg/kg × 2, Day 0 and Day 2). The treatment protocols are similar to those described for B16F10-OVA studies.

#### Combination with Chemotherapy

This was investigated in C57BL/6 mice bearing B16F10 tumors. When tumor sizes reached ∼50 mm^3^, the animals were randomized to receive the following treatments (*n* = 5 for each group): (1) PBS (i.t., 50 μL × 2, Day 0 and Day 2); (2) Carboplatin (i.p., 40 mg/kg, Day 0); (3) Carboplatin (i.p., 40 mg/kg, Day 0) + AnCHNPs (i.t., 200 μg/kg × 2, Day 0 and Day 2). The tumor size and body weight were inspected daily. The tumor was measured in two dimensions with a caliper, and tumor volume was estimated as (length) × (width)^2^/2.

#### Combination with Immunotherapy

This was investigated in C57BL/6 mice bearing B16F10 tumors. When tumor sizes reached ∼50 mm^3^, the animals were randomized to receive the following treatments (*n* = 5 for each group): (1) PBS (i.t., 50 μL, Day 0 and Day 2); (2) Anti-PD-L1 antibodies (i.p., 10 mg/kg, Day −2, 0, 2, and 4); (3) Anti-PD-L1 antibodies (i.p., 10 mg/kg, Day −2, 0, 2, and 4) + AnCHNPs (i.t., 200 μg/kg, Day 0 and Day 2). The tumor size and body weight were inspected every other day.

### Statistical Analysis

All *in vitro* studies were performed in at least triplicate. Half-maximum inhibitory concentration (IC_50_) was determined by Doseresp using Origin 9. All data were represented as mean ± SD. Comparisons of multiple assays were performed using a one-way ANOVA test, and comparisons of two groups were performed using a paired *t* test, with a *p* value of 0.05 or less representing statistical significance.

## Data Availability

The main data supporting the results in this study are available within the paper and its Supporting Information. The raw RNA-sequencing data are available in the NIH GEO database with the accession number GSE208276. Other data generated during the study are available from the corresponding authors upon reasonable request.

## Supporting Information Available

Schematic illustration of AnCHNPs to enhance anticancer immunity, additional physiochemical characterization of nanoparticles, calcium release in solutions and inside cells, effect of degraded AnCHNPs on DC maturation, additional data on the impact of AnCHNPs on immune responses, effect of AnCHNPs on antigen-specific cellular immunity, photos of tumor-bearing mice after receiving different treatment regimens, post-mortem H&E staining results, and flow cytometry gating strategy (PDF)
