# Supplementary material for: Modulation of Dendritic Cell Function via Nanoparticle-Induced Cytosolic Calcium Changes
Source: ACS Nano. 2024 Feb 29;18(10):7618–32. doi: 10.1021/acsnano.4c00550 (PMC10938921; doi:10.1021/acsnano.4c00550)
Supplement: Supplementary file 1 — nn4c00550_si_001.pdf [file nn4c00550_si_001.pdf]

Supporting Information for

Modulation of Dendritic Cell Function *via* Nanoparticle-Induced Cytosolic Calcium Changes

Zhengwei Cao[1], Xueyuan Yang[1], Wei Yang[1], Fanghui Chen[2], Wen Jiang[1], Shuyue Zhan[1], Fangchao Jiang[1], Jianwen Li[1], Chenming Ye[3], Liwei Lang[4], Sirui Zhang[5], Zhizi Feng[1], Xinning Lai[1], Yang Liu[1], Leidong Mao[6], Houjian Cai[3], Yong Teng[2,] and Jin Xie[1,*]*

[1] Department of Chemistry, University of Georgia, Athens, GA 30602, USA

[2] Department of Hematology and Medical Oncology & Winship Cancer Institute, Emory University School of Medicine, Atlanta, GA, 30322, USA

[3] Department of Pharmaceutical and Biomedical Sciences, College of Pharmacy, University of Georgia, Athens, GA 30602, USA

[4] Department of Physiology, Medical College of Georgia, Augusta University, Augusta 30907 GA

[5] Institute of Bioinformatics, University of Georgia, Athens, GA 30602, USA

[6] School of Electrical and Computer Engineering, College of Engineering, University of Georgia, Athens, GA 30602, USA

Supplementary Figures

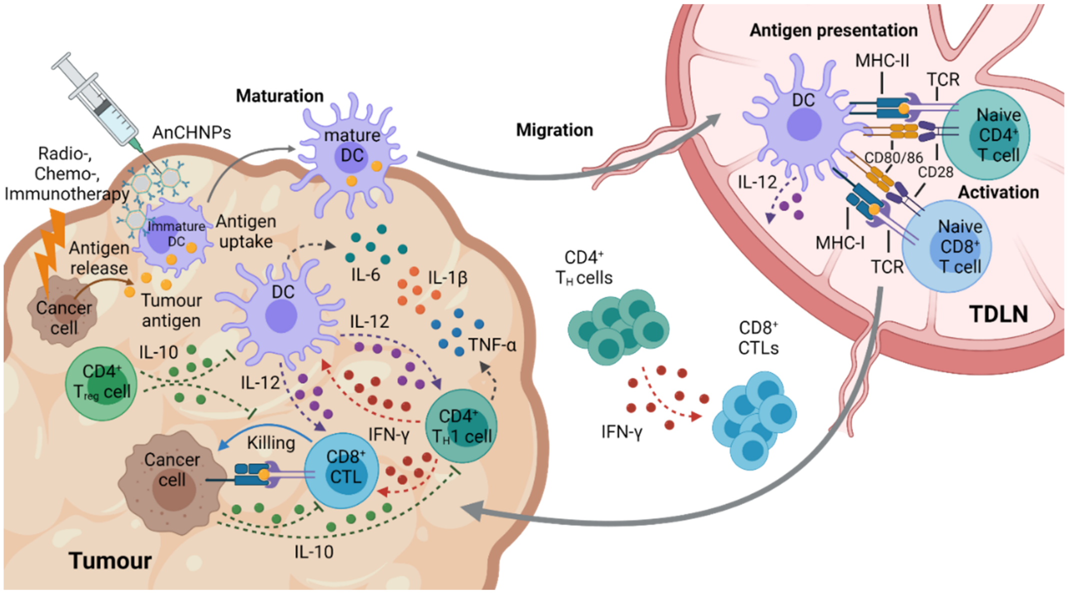

Figure S1. AnCHNPs to boost anti-cancer immunity. AnCHNPs are taken up by DCs, and promote the latter's maturation. The DCs then migration to the secondary lymphoid organs such as the tumor-draining lymph nodes (TDLNs), where they prime the native T cells. The activated DCs also secrete cytokines such as IL-12 that enhance the efficacy of effector T cells. Immunomodulation by AnCHNPs is most effective when they are used following radio- or chemo-therapy, which triggers the release of tumor antigens and DAMPs.

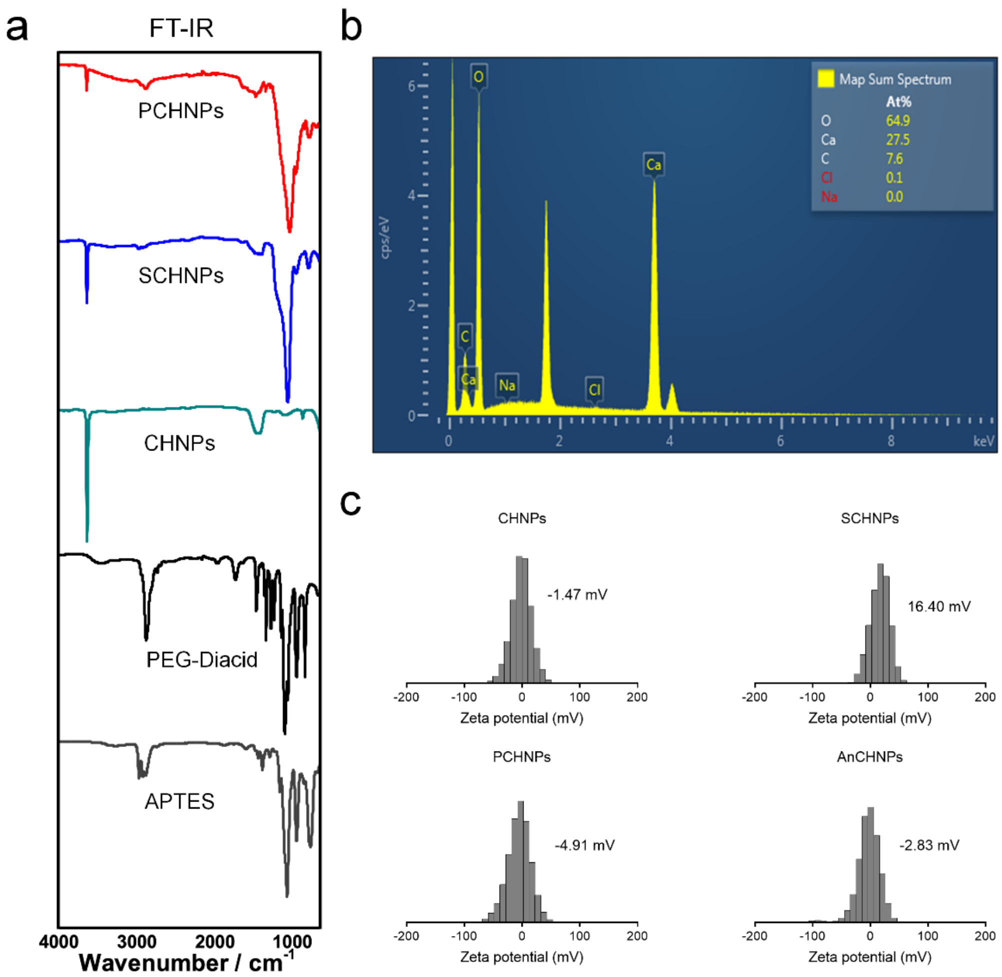

Figure S2. Additional physiochemical characterizations of nanoparticles. These include calcium hydroxide nanoparticles (CHNPs), silica-coated calcium hydroxide nanoparticles (SCHNPs), and PEGylated calcium hydroxide nanoparticles (PCHNPs). **a)** FT-IR spectra of CHNPs, SCHNPs, and PCHNPs. APTES (3-aminopropyl)triethoxysilane), which was used for silica coating, as well as PEG-diacid, which was used for surface PEGylation, were also analyzed. **b)** EDS of CHNPs. The Ca-to-O molar ratio was ~1:2. **c)** Zeta potentials of CHNPs, SCHNPs, PCHNPs, and AnCHNPs, tested in PBS.

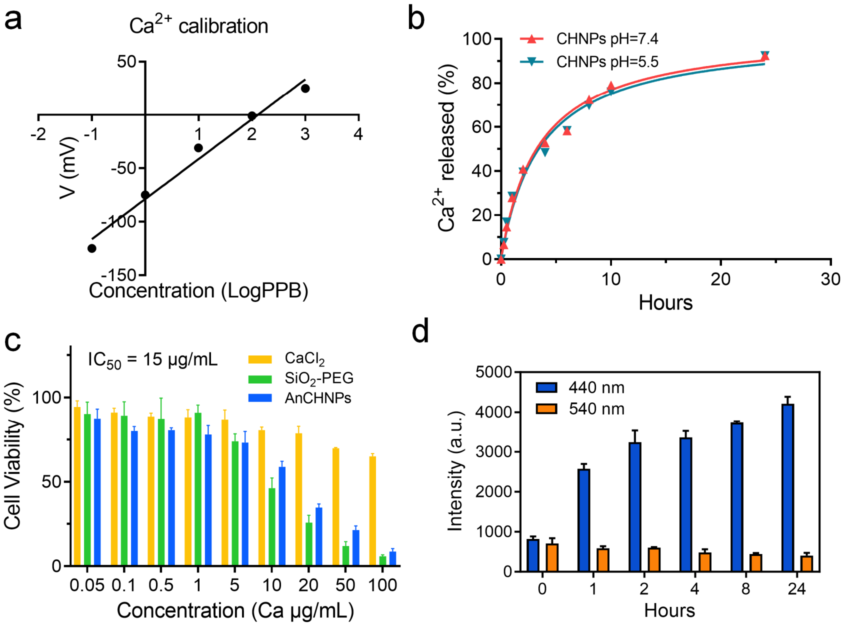

Figure S3. Calcium release in solutions and inside cells. Quantification of calcium levels in solutions was achieved using an ion-selective electrode. *In vitro* quantification was based on a chromogenic calcium indicator, 0-cresolphthalein (OD: 575 nm). **a)** Standard calibration curve for potentiometry measurements, established with calcium salt ($CaCl_2$, 150 ppm and 2000 ppm). **b)** Time-dependent Ca^{2+} release from CHNPs, tested in ammonium acetate buffers at pH 7.4 and 5.5. **c)** Cytotoxicity of AnCHNPs, $CaCl_2$, and aged AnCHNPs, tested with BMDCs using ATPlite-1step luminescence assay. **d)** Lysosomal pH changes after cells being treated with AnCHNPs (5 µg/mL), measured with BMDCs using LysoSensor™ Yellow/Blue DND-160 (PDMPO); the indicator shows predominantly yellow fluorescence (440 nm) in acidic organelles, and in less acidic organelles it shows a stronger blue fluorescence (540 nm).

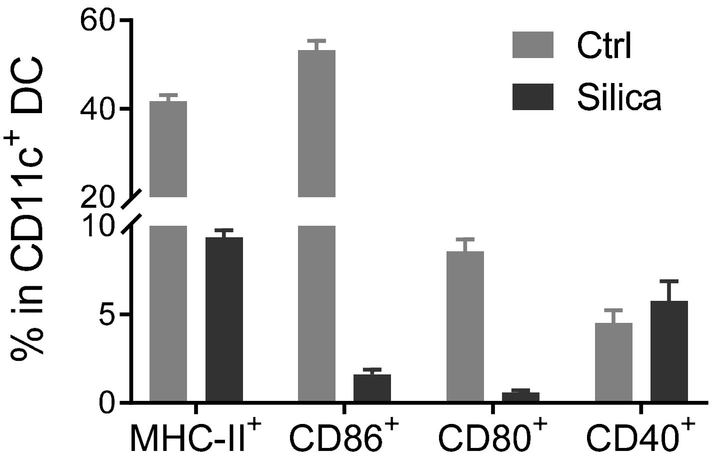

Figure S4. Effect of degraded AnCHNPs on DC maturation. This was investigated by examining maturation markers including MHC-II, CD86, CD80, and CD40 when BMDCs were incubated with aged AnCHNPs.

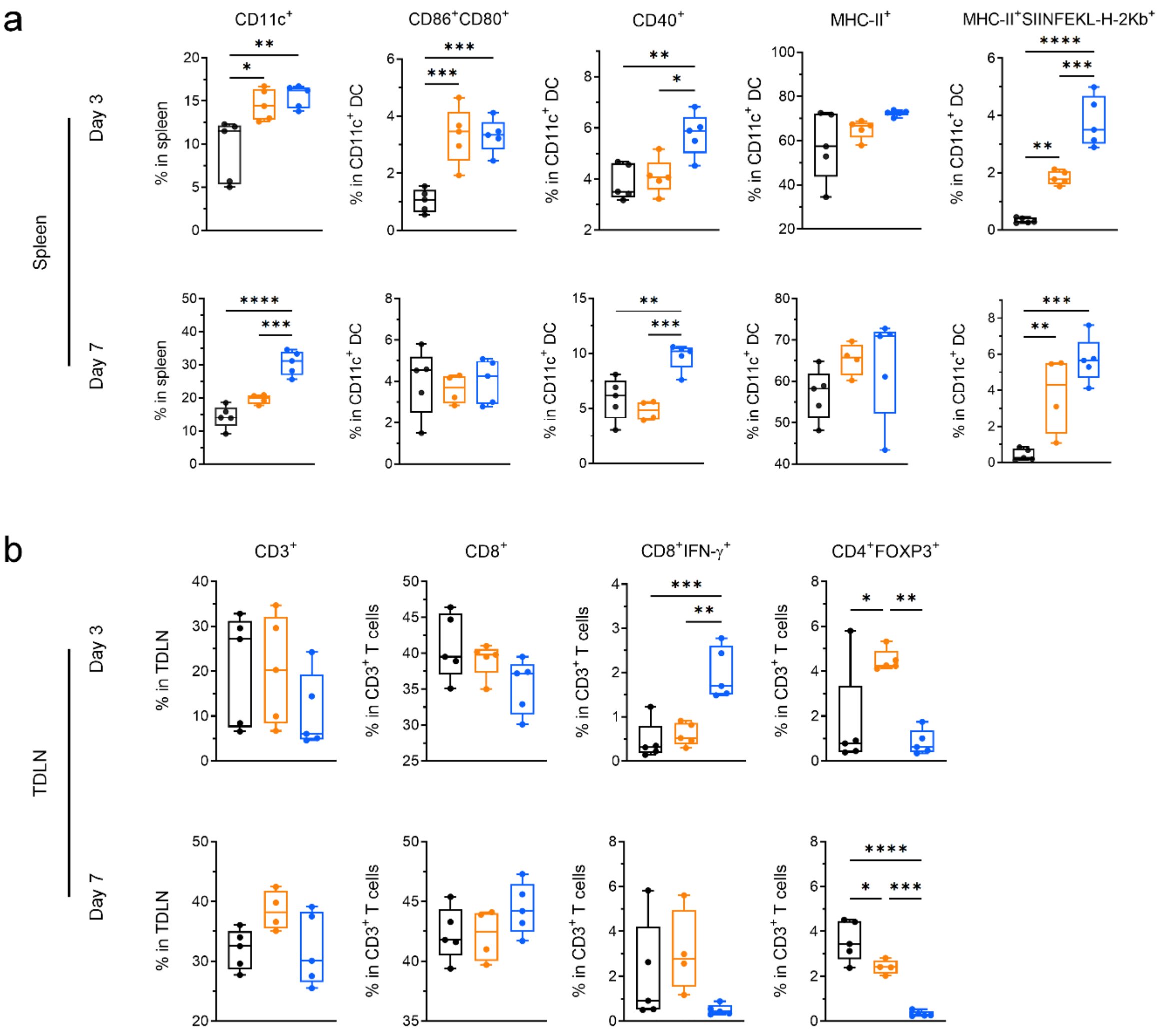

Figure S5. Additional data on the impact of AnCHNPs on immune responses, tested in B16F10-OVA-tumor-bearing C57BL/6 mice. **a)** Populations of $CD86^{+}CD80^{+}$, $CD40^{+}$, MHC-II^{+}, and MHC-II^{+}SIINFEKL-H-$2K^{b+}$ DCs in spleen on Day 3 and 7. **b)** T lymphocyte populations, including CTLs ($CD45^{+}CD3^{+}CD8^{+}$), effector CTLs (IFN-$\gamma^{+}CD45^{+}CD3^{+}CD8^{+}$), and Tregs ($CD45^{+}CD3^{+}CD4^{+}Foxp3^{+}$), in TDLN on Day 3 and 7. *, $p < 0.05$; **, $p < 0.01$; ***, $p < 0.001$; ****, $p < 0.0001$.

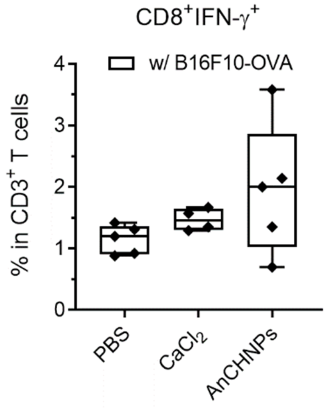

Figure S6. Impact of AnCHNPs on antigen specific cellular immunity. Splenocytes taken from the three treatment groups, AnCHNPs, $CaCl_2$, and PBS, were co-incubated with B16F10-OVA cells for 6 h *ex vivo*; the frequency of IFN-γ^+ CTLs was measured by flow cytometry.

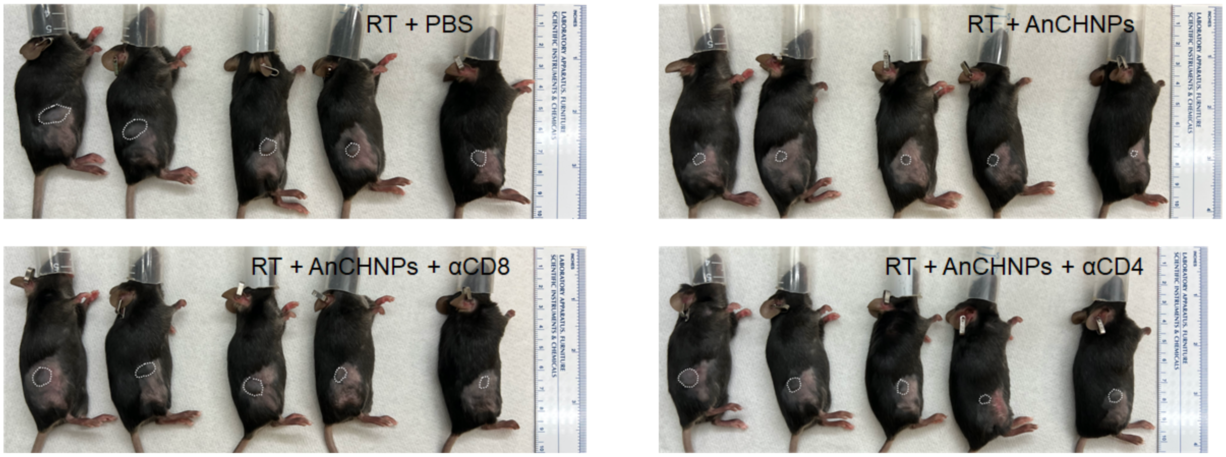

Figure S7. Photos of B16F10 tumor-bearing mice receiving different treatment regimens, taken on Day 14. Radiation therapy (RT, 10 Gy) was given on Day 0 and 2. AnCHNPs were i.t. injected (200 µg/kg) 1 h after each RT session. αCD8 and αCD4 were i.p. injected (10 mg/kg) on Day 0 and Day 4.

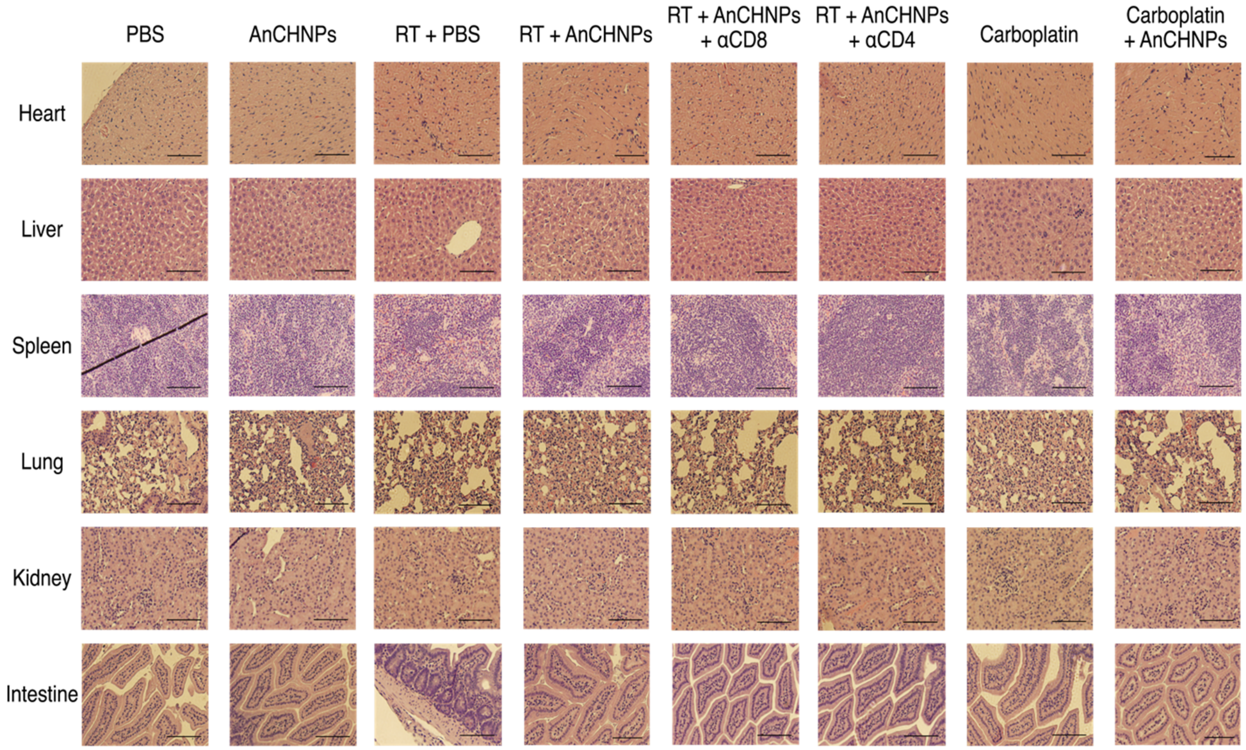

Figure S8. Post-mortem H&E staining of major organ tissues from B16F10-tumor-bearing mice receiving different treatments. These include PBS (i.t., 2 doses on Day 0 and Day 2), AnCHNPs (i.t., 200 µg/kg, 2 doses on Day 0 and Day 2), RT (10 Gy, 2 doses on Day 0 and Day 2) + PBS (i.t., 2 doses on Day 0 and Day 2), RT + AnCHNPs (i.t. administered 1 h post RT), RT + AnCHNPs + αCD8 (anti-CD8 antibodies were i.p. administered at 10 mg/kg, 3 doses on Day -2, Day 0 and Day 2), RT + AnCHNPs + αCD4 (anti-CD4 antibodies were i.p. administered 10 mg/kg, 3 doses on Day -2, Day 0 and Day 2), carboplatin (i.p., 40 mg/kg, one dose on Day 0), and carboplatin + AnCHNPs. Scale bars: 100 µm.

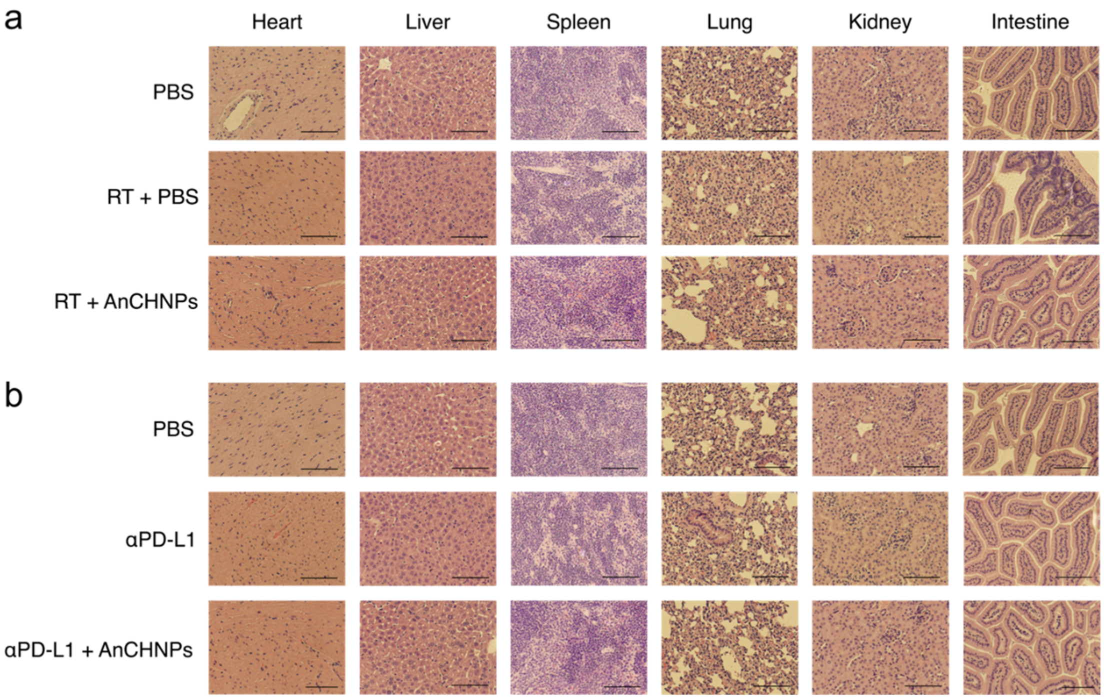

Figure S9. Impact of the combination treatments on major organs, analyzed by histopathology. a) Post-mortem H&E staining of major organ tissues from MB49 tumor bearing mice receiving different treatments. These include PBS (i.t., 2 doses on Day 0 and Day 2), RT + PBS (10 Gy, 2 doses on Day 0 and Day 2, PBS was i.t. administered 1 h post RT), and RT + AnCHNPs (10 Gy, 2 doses on Day 0 and Day 2, AnCHNPs were i.t. administered 1 h after each radiation at a dose of 200 μg/kg). Scale bars: 100 μm. **b)** Post-mortem H&E staining of major organ tissues from B16F10 tumor bearing mice after receiving different treatments. These include PBS (i.t., 2 doses on Day 0 and Day 2), αPD-L1 (i.p. 10 mg/kg, 4 doses on Day -2, 0, 2 and 4), and αPD-L1 + AnCHNPs (AnCHNPs were i.t. administered at a dose of 200 μg/kg on Day 0 and Day 2). Scale bars: 100 μm.

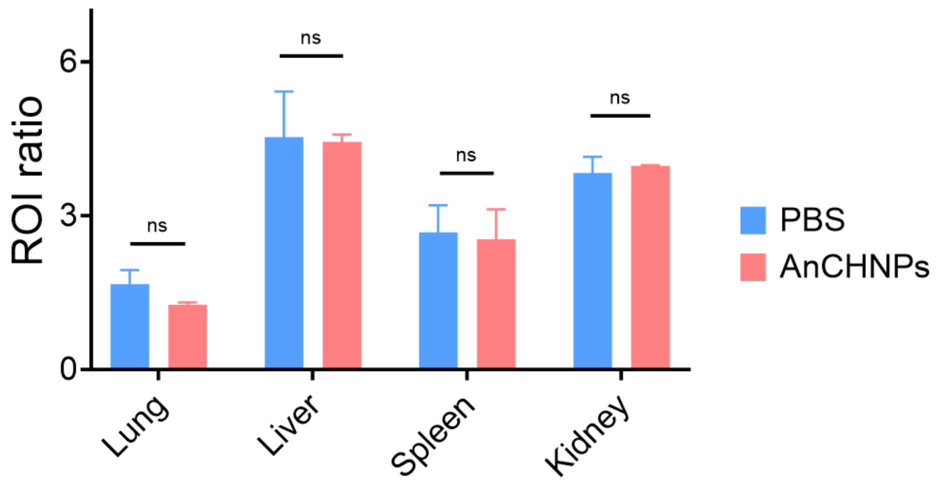

Figure S10. Distribution of nanoparticles (DiR-labeled) in major organs after 24 hours (n=3). Distribution was measured by region of interest (ROI) analysis of photon intensity and the result was normalized to muscle signals. No significant increase in nanoparticle uptake by major organs was observed.

Test	Units	PBS		AnCHNPs		Ref. Interval	
		Ave.	Std.	Ave.	Std.	Low	High
**WBC**	x 10^3/μl	8.33	1.24	9.50	3.96	0.8	10.6
**Neu#**	x 10^3/μl	1.34	0.25	1.47	0.38	0.23	3.60
**Lym#**	x 10^3/μl	6.50	1.18	7.52	3.55	0.60	8.90
**Mon#**	x 10^3/μl	0.36	0.08	0.39	0.20	0.04	1.40
**Eos#**	x 10^3/μl	0.12	0.04	0.09	0.03	0.00	0.51
**Bas#**	x 10^3/μl	0.02	0.01	0.02	0.02	0.00	0.12
**Neu%**	%	16.27	3.09	16.73	3.37	6.5	50.0
**Lym%**	%	53.04	38.81	77.77	5.20	40.0	92.0
**Mon%**	%	4.37	1.12	4.60	2.38	0.9	18.0
**Eos%**	%	1.43	0.40	1.00	0.26	0.0	7.5
**Bas%**	%	0.20	0.10	0.23	0.15	0.0	1.5
**RBC**	x 10^6/μl	8.83	1.42	8.78	1.59	6.50	11.50
**Hgb**	g/dl	14.10	2.23	13.97	2.40	11.0	16.5
**Hct**	%	40.83	6.66	40.03	6.63	35.0	55.0
**MCV**	fl	46.27	0.23	45.67	0.76	41.0	55.0
**MCH**	pg	15.93	0.06	15.87	0.15	13.0	18.0
**MCHC**	g/dl	34.43	0.12	34.77	0.31	30.0	36.0
**RDW%**	%	13.13	0.76	13.13	0.58	12.0	19.0
**Platelet**	x 10^3/μl	798.33	310.86	687.00	287.42	400	1600
**MPV**	fl	5.50	0.10	5.50	0.17	4.0	6.2

Table S1. Complete blood count (CBC) results. PBS or AnCHNPs (200 μg·Ca/kg) were i.t. administered into B16F10 tumor-bearing mice. Blood samples were taken after 7 days (n=3).

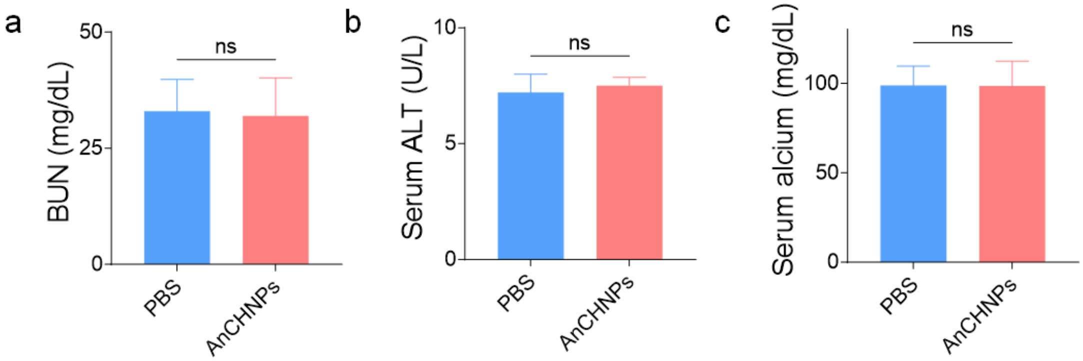

Figure S11. a,b) Serum concentrations of BUN and ALT. Samples were taken 7 days after i.t. injection of PBS or AnCHNPs (n=3). c) Serum concentration of calcium. Samples were taken 24 hours after i.t. injection of PBS or AnCHNPs (n=3)

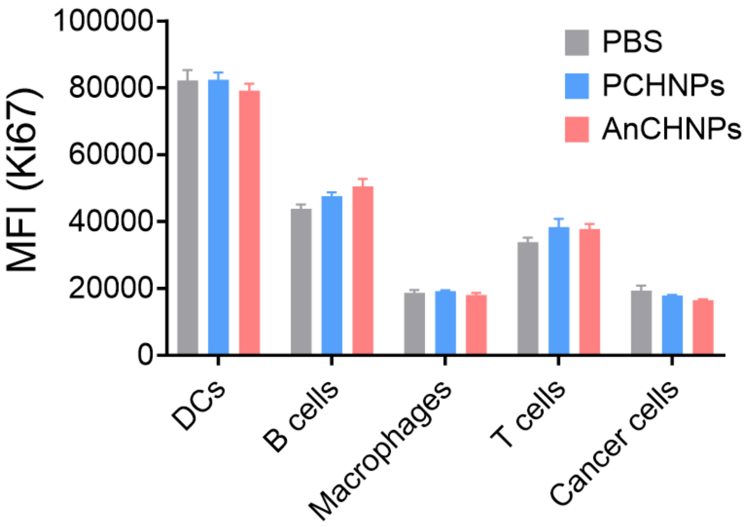

Figure S12. Toxicity of AnCHNPs to cancer cells and tumor-infiltrating immune cells, measured by flow cytometry using Ki67 as a proliferation marker. Samples were taken 24 hours after i.t. administration of AnCHNPs, PCHNPs, or PBS into B16F10 tumor-bearing mice (n=3).

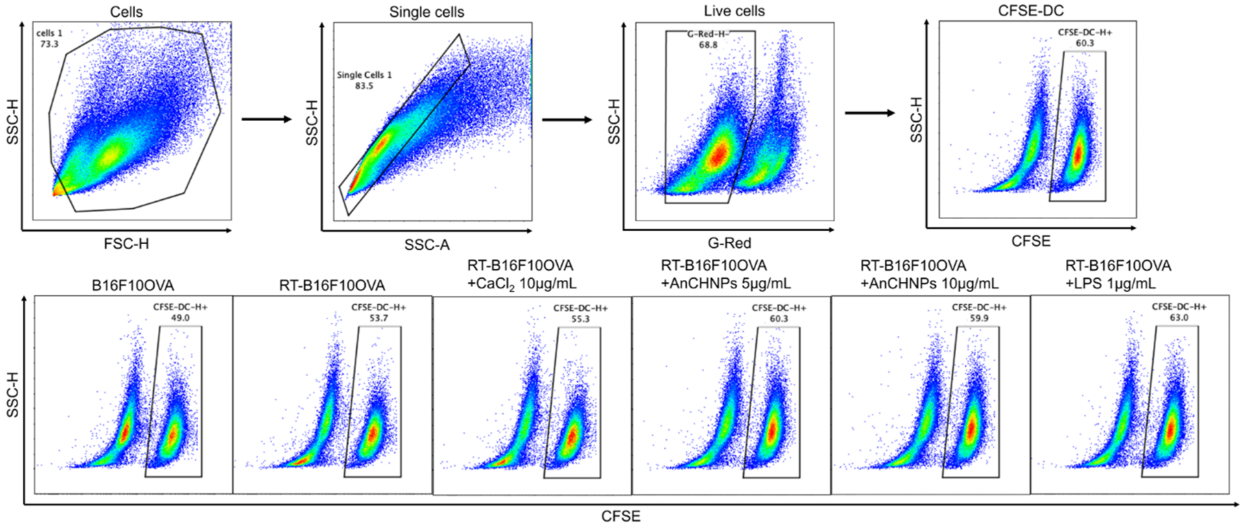

Figure S13. Flow cytometry gating strategy for analyzing DC migration.

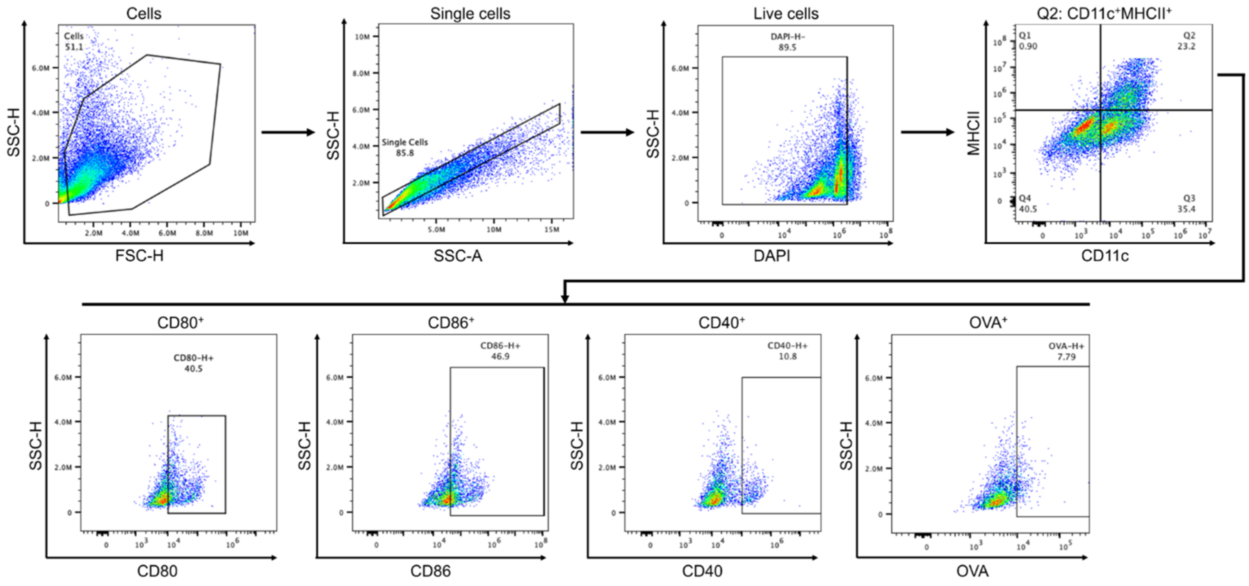

Figure S14. Flow cytometry gating strategy for examining populations of DCs in tumor and TDLNs.

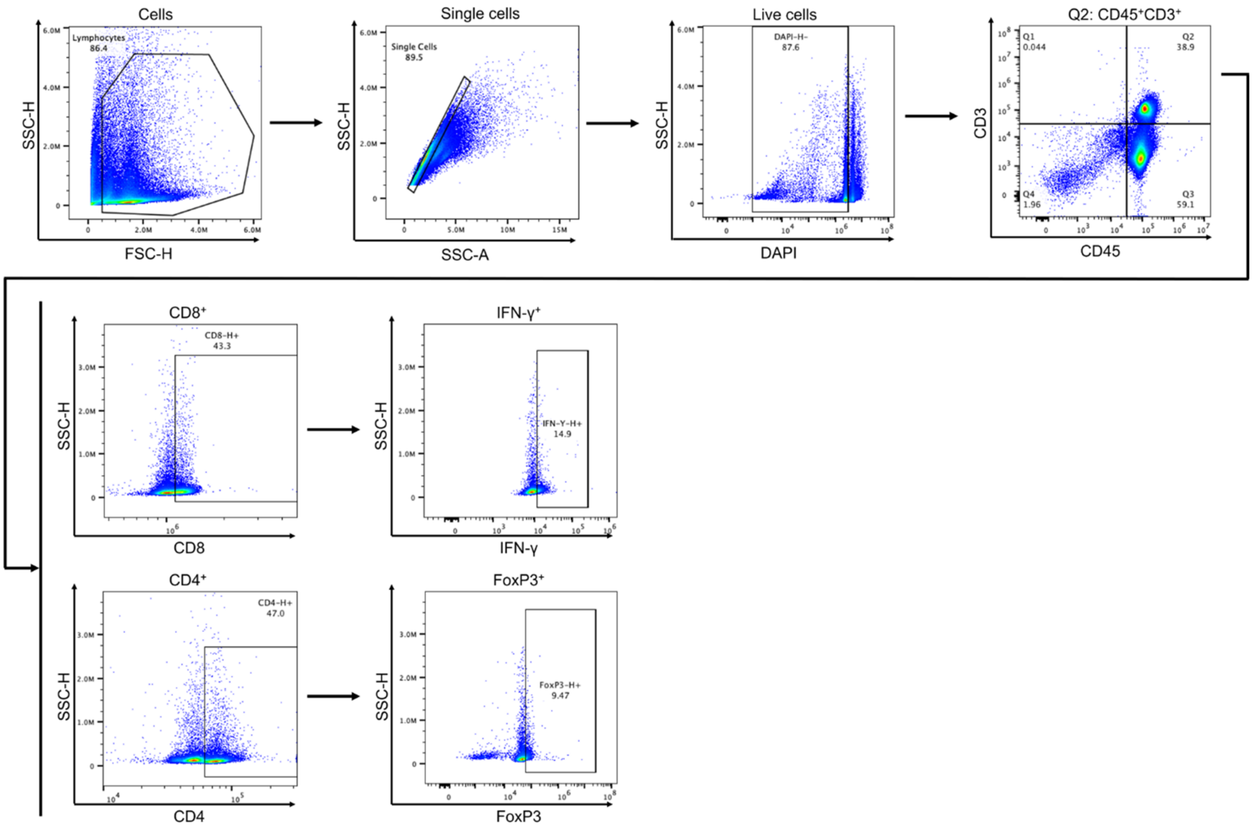

Figure S15. Flow cytometry gating strategy for examining populations of T lymphocytes in tumor and spleen.
